# Role of cold shock proteins B and D in *Aeromonas salmonicida* subsp. *salmonicida* physiology and virulence in lumpfish (*Cyclopterus lumpus*)

**DOI:** 10.1128/iai.00011-24

**Published:** 2024-06-26

**Authors:** Ahmed Hossain, Hajarooba Gnanagobal, Trung Cao, Setu Chakraborty, Joy Chukwu-Osazuwa, Manuel Soto-Dávila, Ignacio Vasquez, Javier Santander

**Affiliations:** 1 Marine Microbial Pathogenesis and Vaccinology Laboratory, Department of Ocean Sciences, Memorial University of Newfoundland, Ocean Science Center, St. John's, Newfoundland, Canada; University of California, Davis, Davis, California, USA

**Keywords:** *Aeromonas salmonicida*, cold shock proteins, marine pathogen

## Abstract

Cold shock proteins (Csp) are pivotal nucleic acid binding proteins known for their crucial roles in the physiology and virulence of various bacterial pathogens affecting plant, insect, and mammalian hosts. However, their significance in bacterial pathogens of teleost fish remains unexplored. *Aeromonas salmonicida* subsp. *salmonicida* (hereafter *A. salmonicida*) is a psychrotrophic pathogen and the causative agent of furunculosis in marine and freshwater fish. Four *csp* genes (*cspB, cspD, cspA*, and *cspC*) have been identified in the genome of *A. salmonicida* J223 (wild type). Here, we evaluated the role of DNA binding proteins, CspB and CspD, in *A. salmonicida* physiology and virulence in lumpfish (*Cyclopterus lumpus*). *A. salmonicida* Δ*cspB*, Δ*cspD*, and the double Δ*cspB*Δ*cspD* mutants were constructed and characterized. *A. salmonicida* Δ*cspB* and Δ*cspB*Δ*cspD* mutants showed a faster growth at 28°C, and reduced virulence in lumpfish. *A. salmonicida* Δ*cspD* showed a slower growth at 28°C, biofilm formation, lower survival in low temperatures and freezing conditions (−20°C, 0°C, and 4°C), deficient in lipopolysaccharide synthesis, and low virulence in lumpfish. Additionally, Δ*cspB*Δ*cspD* mutants showed less survival in the presence of bile compared to the wild type. Transcriptome analysis revealed that 200, 37, and 921 genes were differentially expressed in Δ*cspB*, Δ*cspD*, and Δ*cspB*Δ*cspD,* respectively. In Δ*cspB* and Δ*cspB*Δ*cspD* virulence genes in the chromosome and virulence plasmid were downregulated. Our analysis indicates that CspB and CspD mostly act as a transcriptional activator, influencing cell division (e.g., *treB*), virulence factors (e.g., *aexT*), and ultimately virulence.

## INTRODUCTION

Cold shock proteins (Csp) are small, highly conserved, and structurally related nucleic acid binding proteins that have important roles in the transcriptional regulation of various physiological processes in response to environmental changes, including temperature changes (1 – 4). Csp consist of approximately 65–70 amino acids with a molecular mass of around 7.4 kDa (5 – 8).

Csp have been identified in psychrophiles [e.g., *Arthrobactor globiformis* (9, 10), *Pseudomonas fragi* (11)], mesophiles [e.g., *Escherichia coli*, *Bacillus subtilis* (2, 12 – 16)], thermophiles [e.g., *Bacillus coagulans* (17, 18), *Bacillus caldolyticus,* (7, 19)], and hyperthermophiles [e.g., *Thermotoga maritima*, *Aquifex aeolicus* (7, 20 – 24)]. *E. coli* has nine well studied Csp (CspA to CspI) (2, 16, 23 – 29), and among these cold shock proteins, CspA, CspB, CspG, and CspI are expressed during cold shock (25 – 27, 30).

CspA is an RNA chaperone and plays an important role in transcription and translation (31, 32). At low temperatures (such as 10°C for *E. coli*) CspA binds to ssRNA (single-stranded RNA) and destabilizes secondary structures of mRNAs (31, 33 – 35). CspC and CspE are transcriptional regulators that participate in bacterial cell division, chromosomal condensation, and regulation of stress response proteins (36, 37), and CspD participates in nutrient starvation stress response (36, 38, 39).

Csp also regulate virulence in pathogenic bacteria. For instance, in *Brucella melitensis* and *Salmonella enterica* Δ*cspA*, Δ*cspC,* and Δ*cspE* mutants showed to be attenuated in mice (40, 41). Similarly in *Enterococcus fecalis* Δ*cspR* mutants are attenuated in insects (*Galleria mellonella*) (42), and *Xylella fastidiosa* Δ*csp1* mutants are attenuated in grapevine plants (43). However, the role of Csp in psychrotrophic marine pathogens has not been explored yet.

*A. salmonicida,* a psychrotrophic pathogen, is one of the oldest known fish pathogens. *A. salmonicida,* a Gram-negative, non-motile Gamma proteobacterium, is the causative agent of furunculosis and an important fish pathogen due to its nearly worldwide distribution, broad host range and potentially devastating impacts on wild and farm fish in both fresh and marine water (44 – 49). Lumpfish (*Cyclopterus lumpus*) is a North Atlantic native marine fish utilized as a biological control to delouse the salmon skin and control sea-lice infestations (50, 51). *A. salmonicida* is one of the most frequent pathogens of lumpfish (50) and which is becoming a model to study bacterial infectious diseases in marine cold-water fish (52 – 55).

According to the public genome databases, four cold shock protein genes are present in the genome of *A. salmonicida* J223, *cspA*, *cspB*, *cspC*, and *cspD*. Where CspA and CspC are related to the RNA-binding protein family, and CspB and CspD are related to the DNA-binding protein family (56). CspB has been linked to cold shock adaptation (57), while CspD has been related to stationary-phase stress adaptation and biofilm development (38). In this study, we evaluated the role of CspB and CspD on *A. salmonicida* physiology and virulence in Atlantic lumpfish.

We found that deletion of *cspD* negatively affects the growth of *A. salmonicida* at high temperatures (>28°C), lipopolysaccharide (LPS) synthesis, membrane integrity, and promotes biofilm formation. We observed that the deletion of *cspB* and *cspD* attenuates *A. salmonicida* virulence in lumpfish. Transcriptomics analyses indicated that the essential virulence genes in the chromosome and virulence plasmid were downregulated in *A. salmonicida cspB* and *cspB cspD* mutant strains. These results suggest that CspB and CspD could act as transcription inducers and play a major role in *A. salmonicida* pathogenesis and physiology.

## MATERIALS AND METHODS

### Bacterial growth, culture media, and reagents

The bacterial strains and plasmids used in this study are listed in Table 1. Bacteriological media and components were from Difco (Franklin Lakes, NJ, USA). Antibiotics and reagents were from Sigma (St. Louis, MO, USA). Luria Bertani broth (LB; Tryptone 10 g; Yeast extract 5 g; NaCl 10 g; dH_2_O up to 1L) (58) and Trypticase Soy Broth (TSB; Difco, Franklin Lakes, NJ, USA) were used routinely for bacterial growth. When required, the media was supplemented with 1.5% agar, 10% sucrose, ampicillin (Amp; 100 µg/mL), chloramphenicol (Cm; 25 µg/mL), kanamycin (Km; 50 µg/mL), or Congo red (CR; 0.05%). Bacterial growth was monitored spectrophotometrically and/or by plating. *A. salmonicida* J223 (wild type) (59) was incubated at 15°C with aeration (180 rpm) in a roller drum (TC-7 roller drum, New Brunswick Sci CO, Edison, NJ, USA) until an optical density (O.D.) at 600 nm of ~0.7 Å (mid-logarithmic growth phase) (60, 61). Oligonucleotides were from IDT (Coralville, IA, USA). Restriction endonucleases and T4 ligase were from New England Biolabs (Ipswich, MA, USA). Promega GoTaq Green Master Mix and DNA Polymerase (Madison, WI, USA) were used in all PCR reactions. Agarose 0.8%–1% gel electrophoresis stained with ethidium bromide (0.05%) was used to visualize plasmid, restriction enzyme products, and PCR products. Qiagen QIA spin miniprep kit and MinElute gel extraction kits (Qiagen, Hilden, Germany) were used for plasmid and amplicon purification, respectively.

**TABLE 1:** Plasmid and bacteria used in this study

Strain or plasmid	Genotype or description	Reference or source
*Escherichia coli* strains		
χ289	*E. coli* K-12 wild type A- *ginV44* λ^−^ T3^r^	(62)
χ7213	*E. coli thr-1 leuB*6 *fhuA21 lacY*1 *glnV44 recA*1 Δ*asdA4* Δ (*zhf-2*:: *Tn10*) *thi-1* RP4-2-Tc :: Mu [λpir]; Km^r^	(63)
χ7232	*E. coli endA1 hsdR17* (rK-, mk+) *supE44 thi-1 recA1 gyrA relA1* Δ(*lacZYA*-*argF*) U169 λ*pir deoR* (f80*dlac*Δ(*lacZ*)*M15*)	(64)
Plasmids		
pMEG-375	8,142 bp, Cm, Amp, *lacZ*, R6K *ori*, *mob incP, sacR sacB*	(64)
pEZ151	*ori* pSC101 Gm^r^	(65)
pEZ308	Δ*cspB12*, Cm, Amp	This study
pEZ306	Δ*cspD12*, Cm, Amp	This study
Mutants		
Δ*cspB*	Deleted *cspB* gene from *A. salmonicida* J223	This study
Δ*cspD*	Deleted *cspD* gene from *A. salmonicida* J223	This study
Δ*cspB* Δ*cspD*	Deleted *cspB* and *cspD* gene from *A. salmonicida* J223	This study

### Sequence analysis

BLAST (Nucleotide Basic Local Alignment Search Tool) was done based on *A. salmonicida* wild-type genome (NZ_CP048223) (59). Nucleotide and amino acid sequence alignments were conducted using CLC Genomic Workbench (CLCGW) v20.0.1. Phylogenetic relationship of *cspB* and *cspD* genes of *A. salmonicida* wild type was carried out with MEGA X (66). The web-based interface for ESPript v.2.2 (http://espript.ibcp.fr/ESPript/cgi-bin/ESPript.cgi) (last accessed on 4 January 2024) was used for protein structural-based alignments (67).

The 3D structures of *A. salmonicida* CspB and CspD were *in silico* modeled using position-specific iterative BLAST (PSI-BLAST) alignment and HHpred (68), visualized using VMD v.1.9.4 (http://www.ks.uiuc.edu/Research/vmd/) (last accessed on 4 January 2024) (69).

### Construction and characterization of *A. salmonicida* mutant strains

The recombinant pEZ suicide vectors (Table 1) containing the linked flanking regions were used to create in-frame deletion of *cspB* and *cspD* genes as described previously (59, 65). To prevent polar mutations, the defined deletion mutations included the ATG start codon until one codon before the stop codon but did not include the TAG stop codon. The primers used to construct the suicide vectors are listed in Table S1. Primers F1 and R1 were designed to amplify the upstream gene-flanking regions of the target genes. The downstream-flanking regions of the target genes were amplified by using primers F2 and R2. The flanking regions were ligated using overlapping PCR and cloned into pMEG-375 or pR112 using *SphI* and *XbaI* restriction sites.

To select for defined mutants of *A. salmonicida*, the suicide plasmid was transferred from *E. coli* χ7213 to *A. salmonicida* strains by conjugation. Strains containing single-crossover plasmid insertions were isolated by growing on TSB agar plates supplemented with Cm. The loss of the suicide vector after the second recombination between homologous regions (i.e., allelic exchange) was selected by using the *sacB*-based sucrose sensitivity counter-selection system (70, 71). A^+^ (VapA^+^ or red colonies) Cm^s^ colonies were PCR screened by PCR using primers F1 and R2. The biochemical characteristics of *A. salmonicida* wild type and mutants were analyzed using API20NE (BioMerieux, Marcy-l'Etoile, France), following the manufacturer’s instructions. API 20NE strips containing *A. salmonicida* strains were incubated at 15°C for 48 h, and finally, the results were assessed using APIWEB (BioMerieux).

### Frequency of *vapA* endogenous mutagenesis

To determine the effect of *csp* deletions in the frequency of endogenous *vapA* mutants, 30 µL of fresh bacterial culture was transferred to 3 mL of TSB in 16 mm sterile glass tubes. A triplicate set of each strain were incubated at 15°C, and a second triplicate set was incubated at 28°C. Samples of 100 µL were taken at 14, 17, 20, 41, 45, and 73 h, serially diluted (1:10) and plate quantified onto TSA Congo-red (TSA-CR). The plates were incubated at 15°C for 48 h. After this period, *A. salmonicida* A^+^ and A^−^ (VapA^−^ or white colonies) were quantified. The frequency of A^+^ and A^−^ was calculated according to the formulas:


$$\text{ Frequency of red colonies }\left(A^{+}\right)=\frac{\text{ Number of red colonies }}{\text{ Total colonies }}\text{, and }\;\text{ Frequency of white colonies }\left(A^{-}\right)=\frac{\text{ Number of white colonies }}{\text{ Total colonies }}.$$

### Survival in cold and freezing conditions

Survival of *A. salmonicida* strains to different extreme cold temperatures (−20°C, 0°C, and 4°C) in TSB and seawater was evaluated. *A. salmonicida* strains were grown in 250 mL Erlenmeyer flask containing 30 mL of TSB with aeration (180 rpm) at 15°C until an O.D._600 nm_ of ~0.7 Å (~10^10^ CFU/mL). Bacterial cells were harvested at 4,200 × *g* for 10 min at 4°C, washed, resuspended in 300 µL of phosphate-buffered saline (PBS; 136 mM NaCl, 2.7 mM KCl, 10.1 mM Na_2_HPO_4_, 1.5 mM KH_2_PO_4_ , pH 7.2) (72), serially diluted, and counted by plating onto TSA-CR. An aliquot of 10 µL of the concentrated *A. salmonicida* suspension was transferred to triplicate sets of 990 µL of TSB/seawater. The triplicate sets were independently incubated at −20°C, 0°C, and 4°C for 3 days. Samples were collected daily, serially diluted, and plate counted onto TSA-CR.

### Survival in fresh and seawater

*A. salmonicida* is a widely distributed fish pathogen and it can infect marine and freshwater fish (48, 73). To determine the role of Csp in *A. salmonicida* survival in a free-living stage, we evaluated the survival of the wild-type and mutant strains in freshwater and seawater. Fresh pond water and seawater were collected locally (Logy Bay, NL, Canada), and filter sterilized (0.22 µm). *A. salmonicida* strains were grown with aeration (180 rpm) in 250 mL Erlenmeyer flask containing 30 mL of TSB at 15°C until O.D._600 nm_ of ~0.7 Å (~10^10^ CFU/mL). Bacterial cells were harvested at 4,200 × *g* for 10 min at 4°C and, washed, resuspended in 300 µL of PBS, serially diluted (1:10) and plated counted onto TSA-CR plates. A triplicate set of 16 mm tubes containing 3 mL of freshwater or seawater, were inoculated with 30 µL of fresh *A. salmonicida* (10^8^ CFU/mL) and incubated at 15°C for 32 days. Samples of 100 µL were collected at 3, 7, 14, 21, and 32 days after inoculation, serially diluted (1:10), and plate-counted on TSA-CR plates. The plates were incubated at 15°C for 48 h. Bacterial colony counting was performed according to the established protocols (61).

### Ox bile resistance

Cold shock proteins are related to membrane stress, including resistance to bile salts (74, 75). For instance, CspC and CspE in *S. enterica* serovar *Typhimurium* play an important role in bile salt resistance (41). To evaluate whether CspB and CspD have an impact on membrane stress, we examined bacterial survival in the presence of different concentrations of bile salt. *A. salmonicida* wild type, Δ*cspB*, Δ*cspD*, and Δ*cspB*Δ*cspD* were incubated in 250 mL Erlenmeyer flask carrying 30 mL of TSB with aeration (180 rpm) at 15°C until an O.D._600_ of ~0.7 Å (~10^10^ CFU/mL). Bacterial cells were centrifuged at 4,200 × *g* for 10 min at 4°C, washed, resuspended in 300 µL of PBS, serially diluted, and counted by plating onto TSA-CR supplemented with 1%, 2%, and 5% Ox bile (Sigma-Aldrich, USA). Three biological replicates were used for each condition. The plates were incubated at 15°C for 48 h. Bacterial colony counting was performed according to the established protocols (61).

### LPS profiling

*A. salmonicida* strains were grown in 3 mL of TSB with aeration (180 rpm) at 15°C until O.D._600_ of ~0.7 Å (~10^10^ CFU/mL), and 1 mL was harvested and centrifuged for 10 min at 4,200 × *g* at room temperature. Cell pellets were mixed with 150 µL of LPS buffer-I [10 mL Tris-OH 0.5 M pH 6.8; 8 mL glycerol 10% (vol/vol); 16 mL SDS 10% (vol/vol); 20 mL bromophenol blue 0.05% (vol/vol); 22 mL ddH_2_O]; β-mercaptoehtanol (BME; 50 µL of BME in 950 µL of buffer-I), sonicated (30 kHz; Qsonica sonicator, Q700, Newtown, CT06470, USA), and boiled for 10 min. After cooling down at room temperature, the samples were centrifuged for 15 min at 4,200 × *g* at room temperature. The supernatant was diluted 1:10 into LPS Buffer-II [10 mL Tris-OH 0.5 M pH 6.8; 8 mL glycerol 10% (vol/vol); 20 mL bromophenol blue 0.05% (vol/vol); 22 mL ddH_2_O) and 1 µL of proteinase K (20 mg/mL; Qiagen, Hilden, Germany) was added. The samples were incubated at room temperature for 1 h to digest residual proteins. Aliquots of 7 µL of each sample were loaded in 12% SDS-PAGE gels and separated at 120 V for 2 h using a Mini-PROTEANII Cell electrophoresis apparatus (Bio-Rad, CA, USA). The gel was then incubated in fixative solution [95% ethanol 40% (vol/vol); acetic acid glacial 5% (vol/vol); 530 mL of ddH_2_O] with gentle agitation overnight. The gel was then permeabilized using 0.7% periodic acid in fixative solution for 15 min. After this period, the gel was washed with ddH_2_O with gentle agitation for 10–20 min three times. Then, the gel was stained with silver nitrate solution (2.9 mL NaOH 1M; 2 mL of concentrated ammonium hydroxide, 1 g silver nitrate pre-dissolved in 5 mL of ddH_2_O) for 15 min with gentile agitation at room temperature. After washing three times with ddH_2_O for 5 min, the gel was then covered with develop solution [citric acid 0.005%; formaldehyde 0.05% (vol/vol)]. When the LPS bands were visible and clear enough, stop solution [glacial acetic acid 1% (vol/vol)] was added to the gel and incubated at room temperature for 10 min. Finally, the gel was immersed in a dry solution [glycerol 5% (vol/vol); 95% ethanol 15% (vol/vol)] overnight at room temperature and dried out in ultrafine cellophane paper for later visualization and preservation.

### Outer membrane protein profiles

Sarkosyl-insoluble outer membrane proteins (OMPs) were obtained as previously described (76). Briefly, bacteria were grown in 50 mL TSB in a 250-mL Erlenmeyer flask with aeration (180 rpm) until an O.D._600_ of ~0.7 Å at 15°C. The bacterial culture was then centrifuged at 4,200 × *g* for 10 min at 4°C. The supernatant was eliminated, and the bacterial pellet was resuspended in 50 mL of Tris-OH/EDTA buffer pH 7.4 (20 mM Tris-OH pH 8.0; 1 mM EDTA pH 8.0; 1 mM PMSF). Bacterial cells were lysed by French press (15,000 psi) (Thermo Electron Corporation, USA). To remove cell debris and unlysed cells, the lysed solution was centrifuged at 7,000 × *g* for 10 min at 4°C. The supernatant was transferred to a fresh tube and centrifuged at 16,000 × *g* for 1 h. The supernatant was removed, and the pellet was initially resuspended in 30 µL of Tris-OH (20 mM, pH 8.0), then Sarkosyl solution [20 mM Tris-OH pH 8.0; Sarkosyl (0.5% wt/vol)] was added up to a final volume of 10 mL and incubated overnight in ice. To obtain the OMPs, the suspension was then centrifuged at 16,000 × *g* for 1 h at 4°C. After this, the supernatant was discarded and pelleted OMPs were resuspended in Tris-OH (20 mM pH 8.0). The OMP concentration was normalized to 50 ng/mL by spectrophotometry (Genova Nano, Jenway, Staffordshire, UK) and separated by 12% sodium dodecyl sulfate-polyacrylamide gel electrophoresis (SDS-PAGE). Coomassie blue staining was performed to visualize the OMP.

### SDS-PAGE and Western blot analysis

Protein profiling was conducted for *A. salmonicida* strains according to standard protocols (72). *E. coli* Top10 (77) was used as a control. *A. salmonicida* strains were grown in 3 mL of TSB at 15°C with aeration until an O.D._600_ of ~0.7 Å except for *E. coli* Top10, which was grown in 3 mL of LB at 37°C for 18 h. About 1 mL of culture was collected and centrifuged at 4,200 × *g* at room temperature and washed with PBS once. Bacterial pellets were then resuspended in SDS loading buffer and boiled for 10 min (72). Aliquots of 5 µL were loaded in 12% SDS-PAGE gels and separated at 120 V for 1:40 h using a Mini-PROTEANII Cell electrophoresis apparatus (Bio-Rad, CA, USA). The gel was stained with Coomassie blue [methanol 50% (vol/vol); glacial acetic acid 10% (vol/vol); Coomassie blue 0.125% (wt/vol); ddH_2_O up to 1 L) for 30 min and destained in destined solution [methanol 30% (vol/vol), acetic acid 10% (vol/vol); dH_2_O up to 1 L) (72). Finally, the gels were immersed in a dry solution overnight at room temperature and dried out in ultrafine cellophane paper for later visualization and preservation.

Western blots were performed for the detection of VapA from bacterial cells and purified OMPs. GroEL, a conserved chaperone, was used as a loading control. SDS-PAGE 12% and nitrocellulose membranes (0.2 µm, Bio-Rad, Hercules, CA, USA) were permeabilized in transferring buffer [250 mM Tris-OH; 1.92 M glycine; methanol 20% (vol/vol); ddH_2_O up to 1 L] for 10 min. Proteins were transferred onto nitrocellulose membranes (0.2 µm, Bio-Rad) by using a semi-dry TRANS-BLOTSD apparatus (Bio-Rad) at 20 V for 30 min following the manufacturer’s instructions. Membranes were blocked overnight in blocking buffer [0.5% skim milk in PBS supplemented with 0.05% of Tween 20 (PBS-T)], followed by three washes with PBS-T for 10 min each. Individual membranes were incubated for overnight at room temperature with a primary rabbit polyclonal anti-GroEL antibody (Sigma) (1:5,000) or a rabbit polyclonal anti-VapA antibody (1:5,000) (78) (Santander Lab, custom antibodies collection), washed three times with PBS-T. The membrane was then incubated with a secondary antibody goat anti-rabbit immunoglobulin G (IgG) alkaline phosphatase-conjugated (1:5,000) (Life Technologies, Thermo Fisher Scientific, MA, USA) for overnight with shaking (50 rpm) at room temperature, followed by three washes with PBS-T for 10 min each. The colour was developed by using 500 µL of nitro blue tetrazolium (NBT)−5-bromo-4-chloro-3-indolylphosphate mixture (BCIP) (Thermo Fisher Scientific).

### Biofilm assay

It has been described that Csp regulate the expression of genes related to biofilm (41, 79). To determine the impact of *csp* on *A. salmonicida* biofilm formation, biofilm assays were conducted according to established protocols (80). Briefly, 200 µL of TSB was added into each well of 96-well flat-bottom microtiter polystyrene plate (Becton Dickinson, Franklin Lakes, NJ, USA), and inoculated with 5 µL of *A. salmonicida* mid-logarithmic growth phase culture and incubated for 20 days at 15°C and 28°C. After 20 days post-incubation, the broth was removed, and air-dried overnight, and stained with 200 µL of crystal violet 0.5% per well for 10 min. Then, the crystal violet solution was gently removed, washed with dH_2_O, and air-dried overnight at room temperature. After this period, visualization of the bacteria biofilm was documented in triplicate. Quantification was conducted by adding 200 µL of 95% ethanol to each well to dissolve the attached crystal violet. After 30 min of incubation at room temperature, the absorbance (570 nm) was determined in an enzyme-linked immunosorbent assay plate reader (SpectraMax M5/M5, Molecular Devices Corporation, Sunnyvale, Canada).

Also, the biofilm formation was visualized by confocal microscopy. Sterile rounded microscope slides were placed in 24-well plates and incubated with *A. salmonicida* strains. After 20 days of incubation, the slides were fixed in 4% paraformaldehyde for 12 h at 4°C, washed with PBS, and stained with 5-(4,6-dichlorotriazinyl) amino fluorescein (5-DTAF; 125 µg) and DAPI (4′,6-diamidino-2-phenylindole) according to standards methods (55). Finally, the biofilm was visualized by using a Ti-E confocal microscope (Nikon).

### Bacterial inoculum preparation for animal infection

*A. salmonicida* wild type and mutants were used for infection. A single colony of *A. salmonicida* was grown routinely in 3 mL of TSB at 15°C in a 16‐mm‐diameter glass tube and placed in a roller for 24 h. Then, 300 µL of the overnight culture was added to 30 mL of TSB media in a 250-mL flask and incubated until O.D._600 nm_ of ~0.7 Å (~10^10^ CFU/mL) at 15°C with aeration (180 rpm). The bacterial growth was monitored by spectrophotometry. After this period, the bacterial culture was centrifuged at 4,200 × *g* at 4°C for 10 min. The pellet was washed twice with PBS (72) and centrifuged at 4,200 × *g* at room temperature for 5 min and finally resuspended in 300 µL of PBS. The concentrated bacterial inoculum was serially diluted and quantified by plating onto TSA.

### Lumpfish holding

All the fish procedures were performed at the Joe Brown Aquatic Research Building (JBARB) and the AQ3 biocontainment Cold-Ocean Deep-Sea Research Facility (CDRF), Department of Ocean Sciences (DOS), Memorial University. Animal protocols were approved by the Institutional Animal Care Committee (IACC) (protocols #18-01-JS and #18-02-JS). *C. lumpus* specific-pathogen-free were reared under optimal conditions in 500 L tanks with 95%–100% air saturation and ambient photoperiod, in a flow-through seawater system using filtered seawater, UV-treated, and heated or chilled seawater to maintain an optimal temperature (~10°C). Proper biomass density was maintained in a healthy range (5–30 kg per cubic meter). Lumpfish were fed 3 days per week at a level of 1% body weight per feeding time, using a commercial diet (Skretting, BC, Canada; crude protein 50%, crude fat 18%, crude fiber 1.5%, calcium 3%, and phosphorus 1.4%) until reaching 25 g.

### *C. lumpus* infection with *A. salmonicida*

The virulence assays were performed in the AQ3 biocontainment unit at CDRF, DOS, MUN. Animal protocols were approved by the Institutional Animal Care Committee (IACC) (protocol #18-01-JS). Specific-pathogen-free *C. lumpus* was used with a mean weight of 50 ± 5 g (~6 months old). The animals were randomly assigned to treatment groups of 80 fish each in 500 L tanks. After 2 weeks of acclimation, the fish were intraperitoneally (ip) injected with 100 µL (10^4^ CFU/dose) of *A. salmonicida* (100 LD_50_; lethal dose 50%). Mortality was recorded daily.

### Colonization of *A. salmonicida* in lumpfish tissues

Samples of blood, spleen, liver, head kidney, and brain were taken from five fish per strain at 7 and 10 days post-infection (dpi). The fish were netted and instantly euthanized with an overdose of MS222 (400 mg/L Syndel Laboratories, Vancouver, BC, Canada). Dissected organs were homogenized, serially diluted in PBS (1:10), and plate quantified onto TSA-CR plates. Total bacteria were normalized to 1 g of tissue in accordance with the initial weight of the tissue using the formula (55):


$$CFU*{\;g}^{-1}=\frac{\text{ colony forming units (CFU) }*\text{ original tissue weight }(g)*1{ml}^{-1}*1{\;g}^{-1})}{\text{ original tissue weight }(g)}$$

### Transmission electron microscopy

Lumpfish head kidney tissues were fixed in 10% anhydrous paraformaldehyde (Electron Microscopy Sciences, PA, USA) until the samples were processed at the electron microscopy unit at Memorial University of Newfoundland, St John’s, Canada. The cells were pelleted and resuspended in Karnovsky fixative for 20 min (81) and then washed by using 0.1 M sodium cacodylate buffer pH 7.4 for 5 min and post-fixed in 2% osmium tetroxide (buffered in 50 mM phosphate) for 2 h at room temperature. Then, the fixed cells were washed three times in 50 mM phosphate buffer, washed three times in dH_2_O, and en bloc stained in 0.5% uranyl acetate overnight at 4°C. The pellets were dehydrated in 10 min washes with a sequential acetone series (20, 40, 60, 80, and three times 100%) and infiltrated with Spurr’s resin. Thin sections (70 nm) were cut using an Ultracut R ultramicrotome (Leica Microsystems, Vienna, Austria). Sections were captured on formvar-coated, 300-mesh copper grids, post-stained in uranyl acetate and Sato’s lead citrate, and observed on a Tecnai Spirit TMA with an accelerating voltage of 80 kV.

### RNA extraction for transcriptomics

RNA samples [three biological replicates for each strain (*n* = 3)] were extracted from cultures of *A. salmonicida* strains at 15°C. Wild type and mutant *A. salmonicida* strains were grown under the previously mentioned growth conditions. Once *A. salmonicida* reached the desired growth phase, the cells were centrifuged (4,200 × *g* for 10 min, at 4°C). The cell pellet was utilized for RNA extraction. Total RNA was extracted by using TRIzol (Invitrogen) and purified using RNeasy (QIAGEN), according to manufacturer’s instructions. TURBO DNA-free Kit (Invitrogen) was used to complete the digestion of DNA and to remove remaining DNase and divalent cations such as magnesium and calcium from RNA samples. Purified RNA samples were quantified by using Genova Nano-spectrophotometer (Jenway, Staffordshire, UK) (Table S2) and integrity was determined by agarose gel electrophoresis (Fig. S6A and B) (72).

### Library preparation and RNA-sequencing

For each strain, there were three biological replicates (*n* = 3 per strain). Library preparation and sequencing were done commercially at Genome Quebec. Briefly, RNA quality was evaluated by using NanoDrop spectrophotometer (Thermo Scientific) and a Bioanalyzer 2100 (Agilent). Libraries were generated using the NEBNext Multiplex Oligos for Illumina (Dual Index Primers Set 1; Adapter 1: 3′-AGATCGGAAGAGCACACGTCTGAACTCCAGTCAC-5′; Adapter 2: 3′-AGATCGGAAGAGCGTCGTGTAGGGAAAGAGTGT-5′) and rRNA depleted (1 ng; 5S, 16S, and 23S) using NEBNext rRNA Depletion Kit (Bacteria), according to the manufacturer’s instructions. Sequencing runs were performed on NovaSeq 6000 Sequencer (Illumina) using a NovaSeq 6000 S4 100 bp PE flow cell.

### RNA-seq data analyses

To remove low-quality reads from RNA sequences, paired-end raw reads were mate-paired and filtered by using CLC Genomics Workbench v22.0 (CLCGWB; Qiagen, Hilden, Germany) with default parameters (mate-paired read information, minimum distance = 1; maximum distance = 1000). Next, adapter trimming was performed through CLCGWB utilizing the trim reads tool with default parameters (quality trimming, trim using quality scores, limit: 0.05, and trim ambiguous nucleotides, the maximum number of ambiguities = 2) and to map high-quality reads against *A. salmonicida* wild-type genome NCBI’s Entrez Genome database (NZ_CP048223) using the RNA-Seq analysis tool. To do read mapping and transcript count, the subsequent configurations were used: mismatch cost = 2, insertion and deletion costs = 3, minimum length fraction and minimum similarity fraction = 0.8, the maximum number of hits for a read = 10, and strand-specific = both. The alignment-dependent expectation-maximization (EM) technique based on the RESM, and eXpress approaches were used to do gene expression quantification and normalization of the mapped reads. After normalizing, the counts assigned to each transcript were subjected to compute TPM (transcript per million) reads values by using the trimmed mean of *M* values (TMM) (82). A global correlation analysis was accomplished using log_2_-transformed TPM values (*x* + 1) of each gene of the strains. The Pearson method was used to quantify the correlation. Abundance data analyzation for differential expression was done by the differential expression tool built on a negative binomial general linear model (GLM). Biologically significant differentially expressed genes (DEGs) were picked out with cutoff values of log_2_ fold-change (FC)≥|1| and adjusted false discovery rate (FDR) *P* ≤ 0.05.

### Kyoto Encyclopedia of Genes and Genomes pathway and gene ontology analyses

Cytoscape software (version 3.8.2) with the ClueGO v1.x-v2.5.8 plug-in was used for the gene ontology (GO) and Kyoto Encyclopedia of Genes and Genomes (KEGG) pathway analysis of the differentially expressed genes. Functionally grouped GO annotation networks were created by the ClueGO plug-in for all the differentially expressed genes. GO categories were separated into biological process, molecular function and cellular components and GO term fusion, medium network specificity, were selected. GO terms and KEGG pathways with a *P ≤ 0.05* were treated as a significant and Kappa Score Threshold = 0.4 were considered for the analysis. Protein-protein interaction was done by using STRING version 12.0 (https://string-db.org/cgi/input?sessionId=bAOkt9BguVNC&input_page_show_search=on).

### Statistics

Data visualization and statistical analysis were done by using Prism package v7.0 (GraphPad Software, La Jolla, CA, USA). To get significant variations, a nonparametric one-way ANOVA Kruskal-Wallis test was conducted, followed by Dunn’s multiple comparison *post hoc* test and *P* ≤ 0.05 was considered to indicate statistical significance. For getting differences in survival rates post-infection, the Kaplan-Meier estimator was applied. To do comparison trends in survival curves, the Log-rank test was used. To assess tissue colonization, a one-way ANOVA combined with a nonparametric Kruskal-Wallis test was utilized. Subsequently, Dunn’s multiple comparison *post hoc* analysis was applied to identify significant differences in colonization between the wild type and mutant strains.

## RESULTS

### *A. salmonicida* CspB and CspD analysis

The amino acid sequence alignment revealed that all the residues required for a functional cold shock protein are very conserved in the Csp analyzed. Cold shock proteins B and D of *A. salmonicida* contained the canonical nucleic acid-binding sequence motifs RNP1 (K/R-G-F/Y-G/A-F-V/I-X-F/Y) and RNP2 (L/I-F/Y-V/IG/ K-N/G-L) required for a functional Y-box DNA binding domain (2, 83 – 86). Both CspB and CspD motifs RNP1 and RNP2 were present in β2 and β3 barrel (Fig. S2A and S3A).

Phylogenetic relationship analysis of *A. salmonicida cspB* showed a closed phylogenetic relationship with *Mycobacterium tuberculosis* and a distant relationship with *S. enterica* (Fig. S2B). In addition, it was found that *cspD* from *Pasteurella multocida* has a close relationship with *A. salmonicida* and a far phylogenetic relation with *Edwardsiella ictaluri* (Fig. S3B). The 3D structure has shown that both CspB and CspD contained β-barrel in their structure (Fig. S2C and S3C).

### Construction and phenotypic characterization of the *A. salmonicida cspB* and *cspD* mutant strains

**Fig 1:**
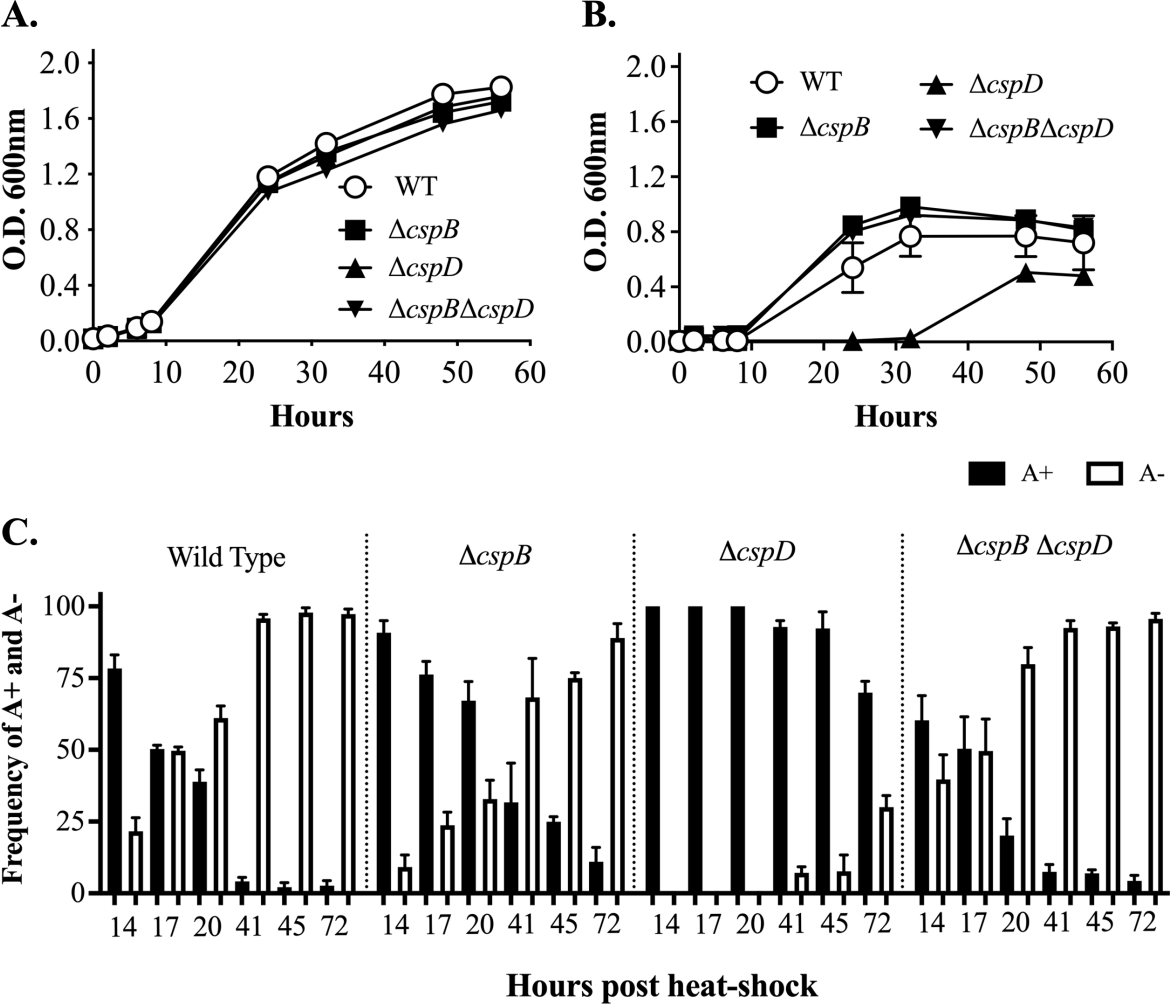
Effect of temperature on *A. salmonicida* growth and endogenous mutagenesis. (A) Growth of *A. salmonicida* at 15°C. (B) growth of *A. salmonicida* at 28°C. (C) frequency of A+ (black bars) and A− (white bars) colonies from *A. salmonicida* cultures at 15°C and 28°C.

*A. salmonicida,* 660 bp of *cspB* and 219 bp of *cspD* genes were deleted to construct Δ*cspB* and Δ*cspD* mutants, respectively (Fig. S1A and B). The construction of Δ*cspB* and Δ*cspD* mutants was performed using the plasmids pEZ308 and pEZ306 suicide vectors (Table 1). *A. salmonicida* Δ*cspB* and Δ*cspD* mutants were selected on TSA plates supplemented with 10% sucrose and confirmed through PCR using the primers F1 and R2 for each mutation (Fig. S1C; Table S1). Cold shock proteins have a role in growth (1); thus, we checked the growth phenotype at 15°C and 28°C. There were no differences in growth recorded at 15°C. However, at 28°C, Δ*cspD* mutants showed slower growth compared to the wild type and other mutant strains. These results suggest that the CspD plays a role in *A. salmonicida* growth at high temperatures (Fig. 1A and B). The biochemical analysis using API20NE showed some differences between the wild type and mutants. *A. salmonicida* Δ*cspB* and Δ*cspB*Δ*cspD* showed negative reaction for arginine dihydrolase and positive reaction for glucose assimilation, mannitol assimilation, and N-acetyl-glucosamine assimilation. *A. salmonicida* Δ*cspD* mutant showed a negative reaction for acidification, maltose assimilation, and malate assimilation (Table S3). Complementation of the mutant was attempted using several plasmid vectors with high, medium, and low copy numbers, but despite our efforts *A. salmonicida* did not accept these plasmids (data not shown).

### Frequency of A− and A^+^ *A. salmonicida* strains

*A. salmonicida* genome is rich in insertion sequences (ISs), which are thermo-inducible (87). It has been shown that *vapA* and *abcA* encoding genes for protein A (VapA) and the ATP-binding cassette transport protein, respectively, could be interrupted by thermal inducible ISs (44, 87). We evaluated the frequency of endogenous mutagenesis of the *vapA* gene in the wild type and *csp* mutant strains during growth at 28°C to determine whether cold shock proteins could influence ISs activity. We found that the frequency of endogenous mutagenesis in the gene *vapA* was initially detected after 12 h at 28°C and increased up to almost 100% A^−^ phenotype after 24 h (Fig. 1C). The frequency of endogenous mutagenesis of ΔcspB and ΔcspBΔcspD were the same as the wild type. However, ΔcspD showed delayed endogenous mutagenesis kinetics compared to other strains due to a slower growth at 28°C (Fig. 1C).

### Survival in low temperature and freezing conditions

**Fig 2:**
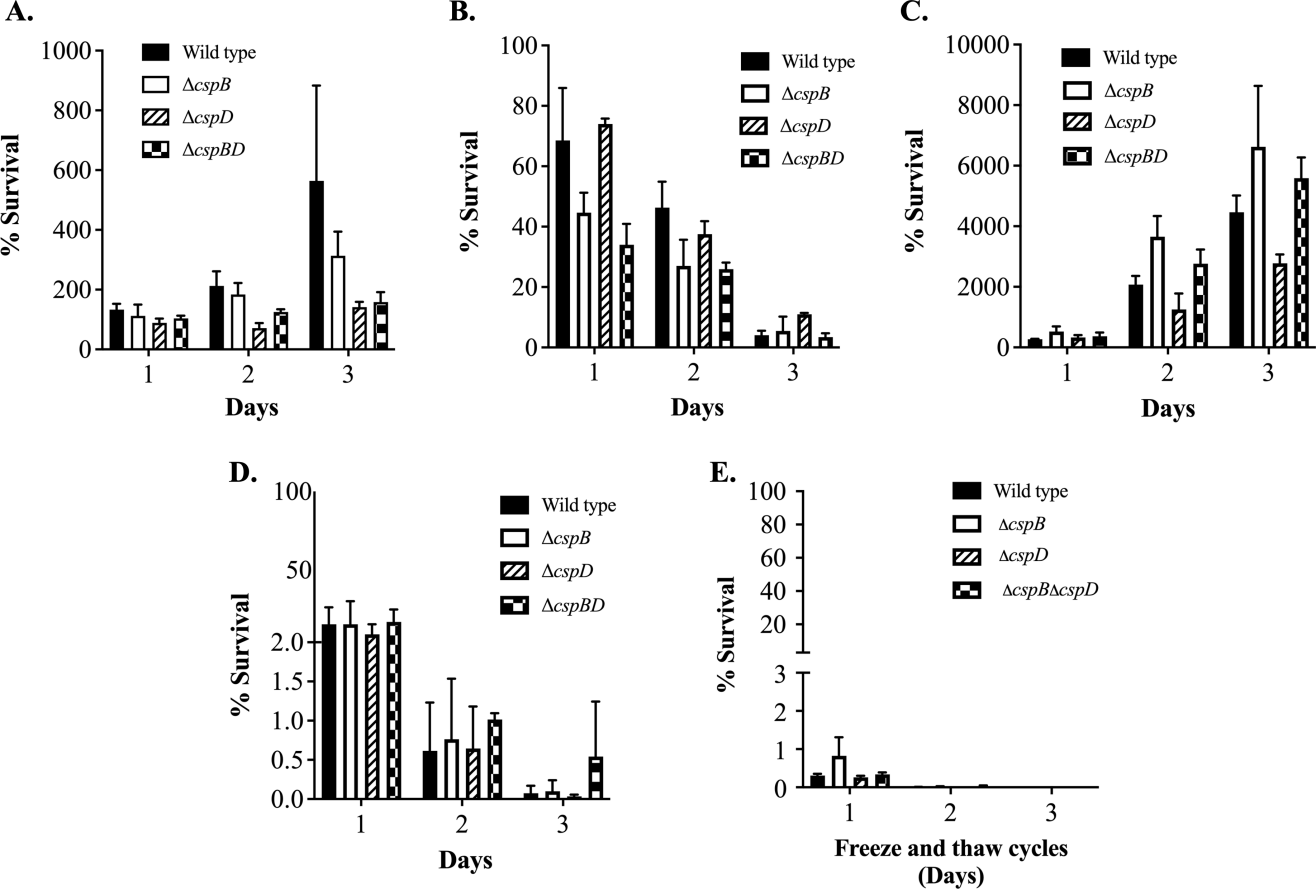
*A. salmonicida* survival at different temperatures in seawater, freshwater, and TSB culture media. *A. salmonicida* wild type and mutants (three biological replicates for each strain) were incubated at low temperatures (0°C and 4°C) and freezing conditions (−20°C) for 3 days. Samples were collected every 24 h and transferred to the TSA-CR plates and incubated at 15°C. (A) Survival rates of wild type, mutants in TSB at 0°C. (B) Survival rates of wild type, mutants in TSB at 4°C. (C) Survival rates of wild type, mutants in seawater at 0°C. (D) Survival rates of wild type, mutants in seawater at 4°C. (E) Survival rates of wild type, mutants at −20°C (*P ≤ 0.05, **P ≤ 0.01, ***P ≤ 0.001, ****P ≤ 0.0001, the nonparametric Kruskal-Wallis test, followed by Dunn’s multiple *post hoc* test, was done to determine the significant differences).

It has been reported that Csp play a critical role in adaption to cold shock (4). Therefore, here we evaluated the survival of *A. salmonicida csp* mutants after exposure to cold temperatures at 0°C and 4°C in TSB and seawater. In TSB at 0°C and 4°C, the survival rate of ΔcspD was the lowest compared to the wild type (*P ≤* 0.05) (Fig. 2A and B). When the survival test was conducted in seawater, survival of ΔcspBΔcspD was lower than the other strains at 0°C, and at 4°C the survival of ΔcspD was lower compared to the wild type and other mutants (Fig. 2C and D). Survival in seawater was lower compared to survival in TSB for all the strains at both 0°C and 4°C (Fig. 2A through D). Although mutants and wild type survived on the first day of freezing condition, no survivor cells were recovered after the second day of treatment at −20°C (Fig. 2E).

### Survival in seawater and freshwater

In freshwater and seawater, there was no significant difference in survival between the mutants and wild type at 15°C. However, after 32 days, survival in seawater ofΔ*cspD* mutant was lower than survival in freshwater (Fig. S4A and B).

### Survival in Ox bile

Evident growth of mutants and the wild type was observed on 5%, 2%, and 1% ox bile-containing TSA plates. Survival of Δ*cspB* and Δ*cspD* was higher than the wild type, and survival of Δ*cspB*Δ*cspD* was lower than the other mutants and the wild type in all ox bile concentrations (Fig. S5). These results suggest that both cold shock proteins are important to survive in the presence of bile salt.

### *A. salmonicida* O-PS (O-polysaccharide) analysis

**Fig 3:**
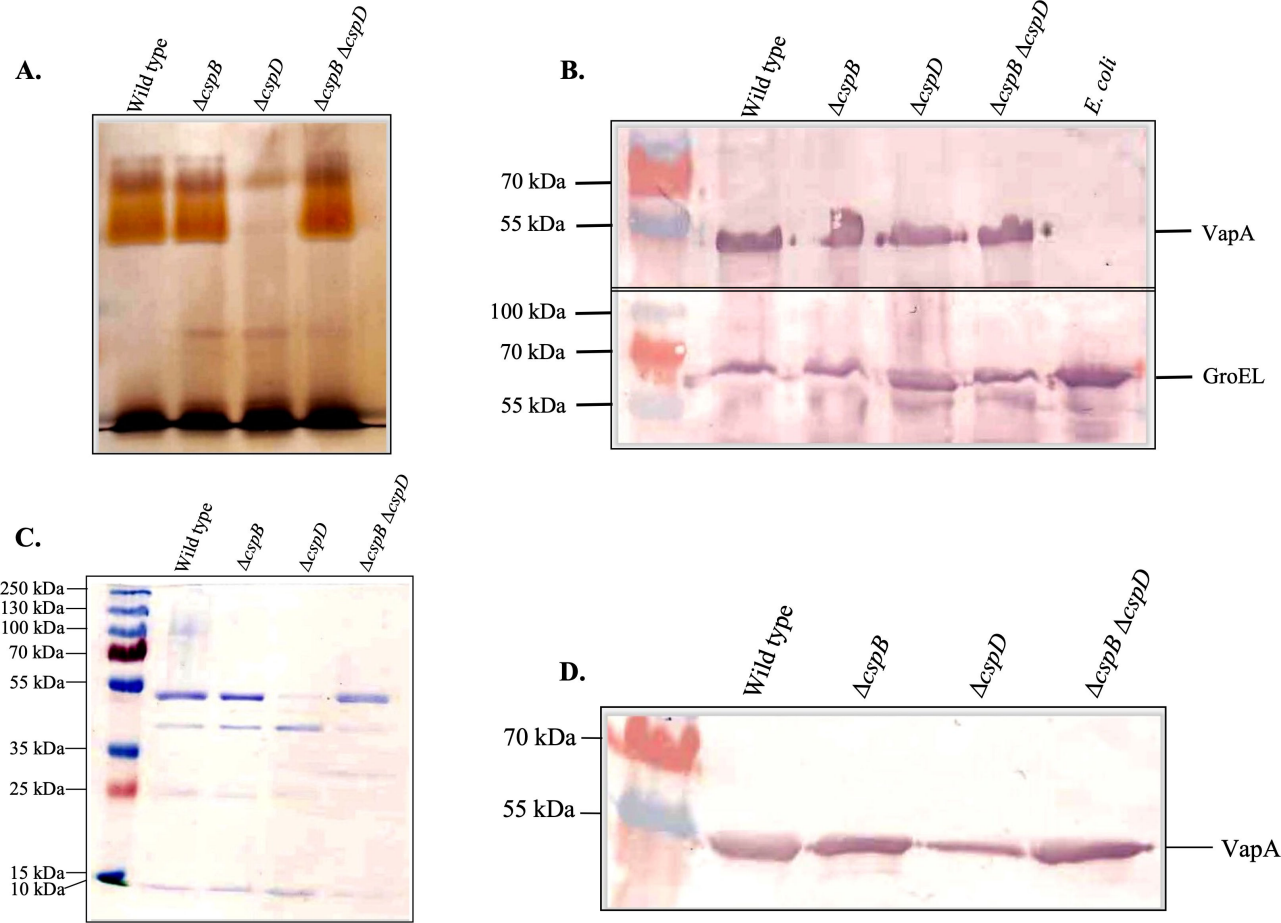
Lipopolysaccharide (LPS) and outer membrane protein (OMP) profiles of *A. salmonicida*. (A) LPS profiles of *A. salmonicida* wild type, Δ*cspB*, Δ*cspD*, and Δ*cspB* Δ*cspD* strains. (B) VapA and GroEL Western blot analysis in *A. salmonicida* wild type, Δ*cspB*, Δ*cspD*, and Δ*cspB* Δ*cspD* strains whole cells. (C) OMP profiles of *A. salmonicida* wild type, Δ*cspB*, Δ*cspD*, and Δ*cspB* Δ*cspD* strains. (D) VapA and GroEL Western blot analysis in *A. salmonicida* wild type, Δ*cspB*, Δ*cspD*, and Δ*cspB* Δ*cspD* OMPs.

Deletion of *cspB* or *cspD* and *cspB cspD* did not affect LPS synthesis. Though deletion of *cspD* gene caused the disruption in O-PS synthesis in *A. salmonicida*, the LPS-core seems unaffected. This indicated that *cspD* could participated in O-PS synthesis regulation (Fig. 3A).

### Outer membrane protein profile of *A. salmonicida*

Outer membrane protein profile for all the mutant strains was shown to be considerably like the wild type with the exception of Δ*cspD. A. salmonicida* Δ*cspD* showed a disruption of an outer membrane protein of approximate molecular weight of 53 kDa (Fig. 3C). However, no changes in VapA protein synthesis were observed after the deletion of the cold shock protein genes (Fig. 3B and D).

### Impact of *csp* deletion on biofilm formation

**Fig 4:**
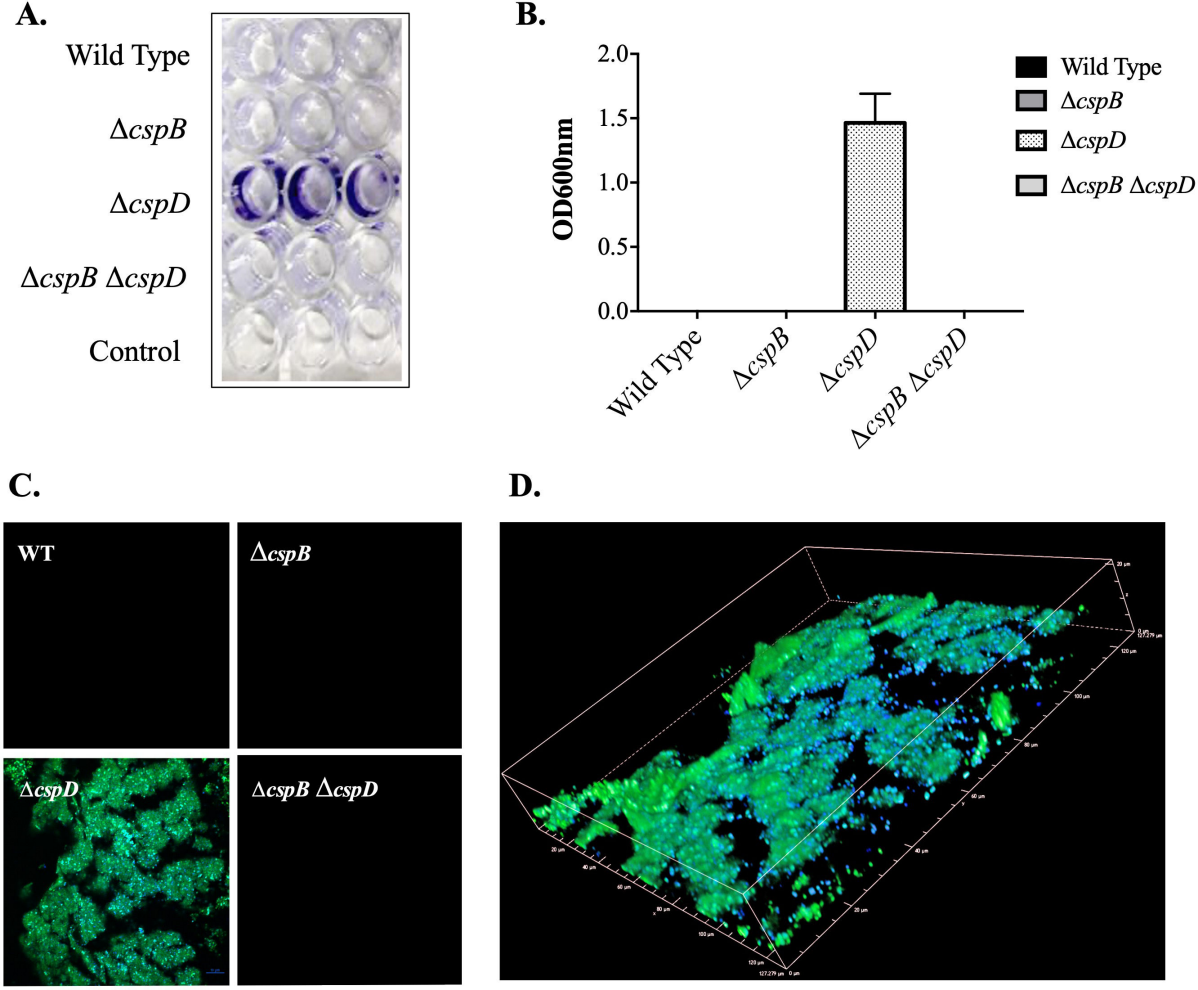
*A. salmonicida* biofilm formation test. (A) Biofilm formation at 15°C after 21 days stained with crystal violet in 96-well plates. (B) Biofilm quantification at OD 570 nm. (C) Confocal microscopy of *A. salmonicida ∆cspD* biofilm (1,000×). (D) 3D confocal microscopy image of *∆cspD* biofilm.

*A. salmonicida* Δ*cspD* formed a strong biofilm (Fig. 4A and B). In contrast, wild type and Δ*cspB*, Δ*cspB*Δ*cspD* mutants did not form biofilm. The crystal violet stain captured by the Δ*cspD* biofilm was nearly 1.4 Å, while there was no detectable absorbance for wild type *A. salmonicida* and Δ*cspB* and Δ*cspB*Δ*cspD* mutants. Under the confocal microscopy, a very thick biofilm Δ*cspD* was revealed (Fig. 4C and D). These results suggested that *cspD* is involved in biofilm regulation in *A. salmonicida*.

### Fish survival and tissue colonization

**Fig 5:**
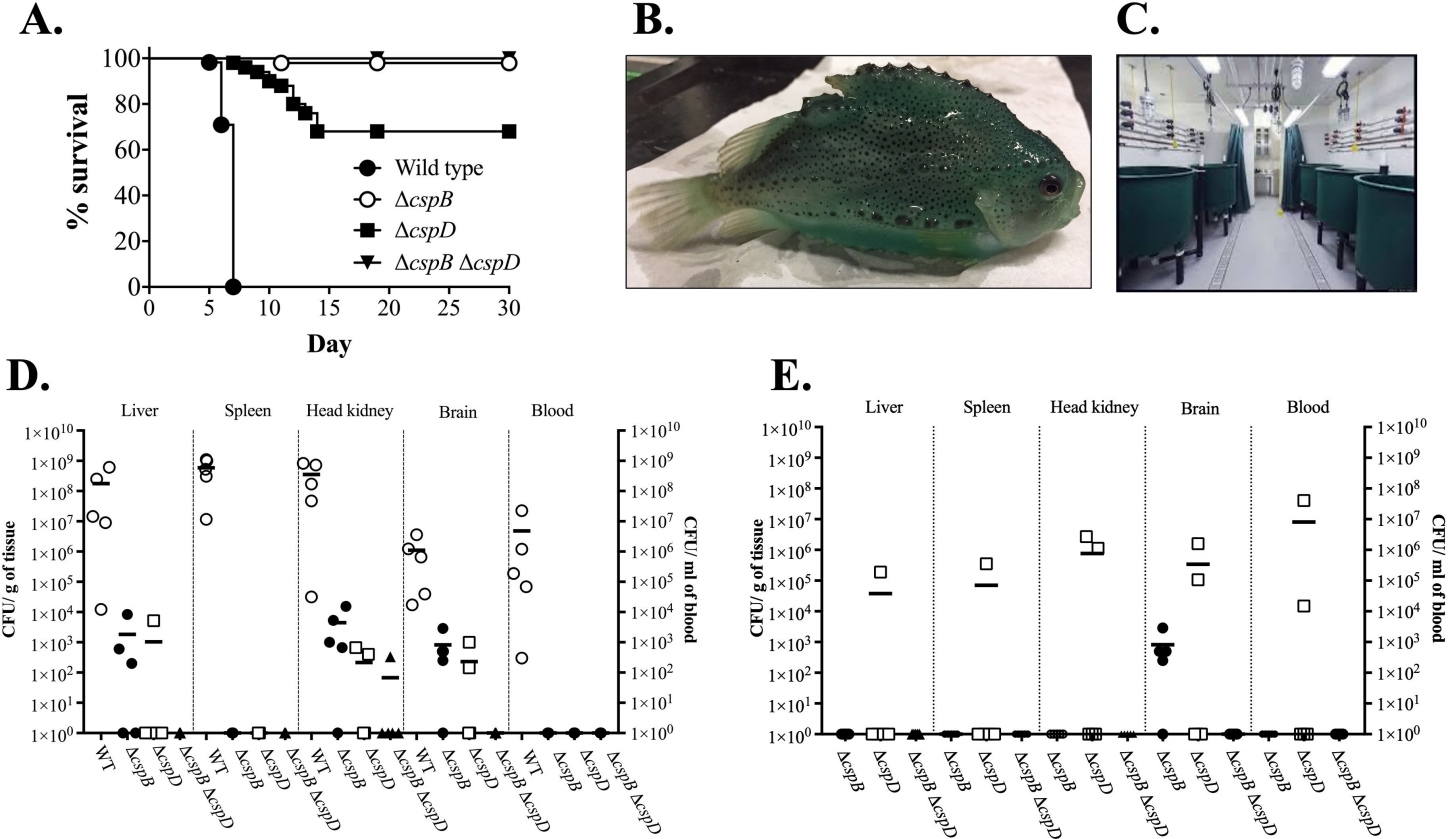
Virulence and tissue colonization of *A. salmonicida* wild type, Δ*cspB*, Δ*cspD*, and Δ*cspB* Δ*cspD* strains in lumpfish. (A) Survival of lumpfish after *A. salmonicida* i.p. injection (~10^4^ CFU/dose) (****P ≤ 0.0001). (B) Healthy lumpfish used in this study*,* ~25 g. (C) Infection room and tanks setup the AQ3 faculty. (D) *A. salmonicida* tissue colonization after at 7 dpi. Significant differences (*P ≤ 0.05) in the bacterial loads of *A. salmonicida* mutant strains compared to the wild type were observed in the spleen, as determined by the nonparametric Kruskal-Wallis test. (E) *A. salmonicida* tissue colonization after at 10 dpi.

Csp are important for bacterial pathogenicity in several species, for instance, *S. enterica*, *E. fecalis*, *B. melitensis*, and *Staphylococcus aureus* mutants of *csp* are attenuated (41, 42, 88, 89). Virulence of *A. salmonicida* wild type and *A. salmonicida* Δ*cspB*, Δ*cspD*, Δ*cspB*Δ*cspD* were evaluated in lumpfish (50–55 g) by i.c. injection. Mortality of fish infected with the wild type started at 5 dpi, gradually increased, and reached 100% at 7 dpi (Fig. 5A through C). In contrast, fish infected with Δ*cspD* and Δ*cspB* started to die at 7 and 11 dpi, respectively, and survival was stabilized at 14 and 19 dpi, respectively. The RPS (Relative Percentage of Survival) recorded for theΔ*cspB*, Δ*cspD*, Δ*cspB*Δ*cspD* groups were 94%, 66%, and 100%, respectively (Fig. 5A). Significant lowest survival (*P* ≤ 0.0001) was noticed in Δ*cspD* infected group, whereas no mortality was observed in Δ*cspB*Δ*cspD* infected groups. *A. salmonicida* wild type, Δ*cspB*, Δ*cspD*, and Δ*cspB*Δ*cspD* infection kinetics were determined in liver, spleen, head kidney, brain, and blood in lumpfish at 7 and 10 dpi. Lumpfish infected with *A. salmonicida* wild type showed significantly higher bacterial loads in all sampled tissues compared to the animals infected with *csp* mutants. For instance, at 7 dpi, the bacterial load in liver, spleen, head kidney, brain, and blood of fish infected with the wild type of strain were 1.7 × 10^8^ CFU/g, 5.9 × 10^8^ CFU/g, 3.5 × 10^9^ CFU/g, 1.1 × 10^6^ CFU/g, and 4.8 × 10^6^ CFU/mL, respectively (Fig. 5D). In contrast, after 7 dpi, *A. salmonicida* mutant strains were mostly cleared from the spleen and blood. Few Δ*cspB* and Δ*cspD* colonies were detected in the liver, head kidney, and brain at 7 dpi. At 10 dpi, only Δ*cspD* was detected in all sampled organs, and Δ*cspB* was detected only in the brain. Very few Δ*cspB*Δ*cspD* colonies were detected in head kidney after 7 dpi (Fig. 5D and E). These results suggest that CspB and CspD play a pivotal role in *A. salmonicida* virulence.

### Transmission electron microscopy

**Fig 6:**
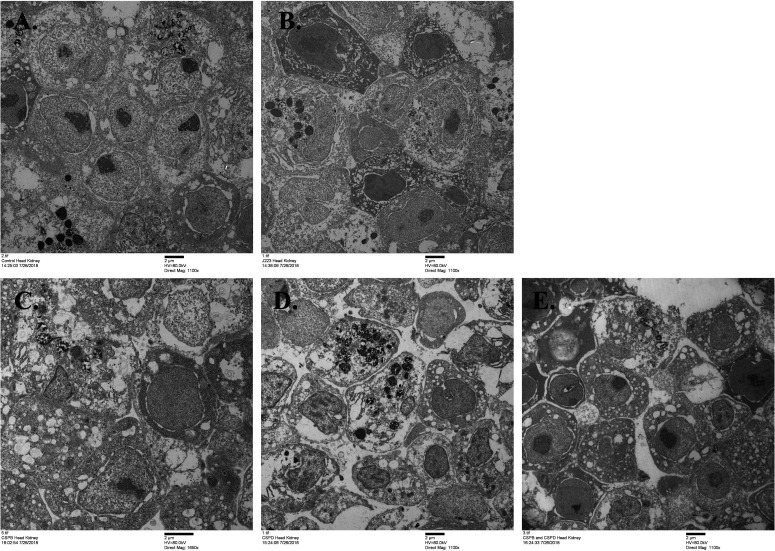
Transmission electron microscopy of lumpfish head kidney infected with *A. salmonicida* at 7 dpi. (A) Control non-infected. (B) *A. salmonicida* wild type. (C) *A. salmonicida ∆cspB*. (D) *A. salmonicida ∆cspD*. (E) *A. salmonicida ∆cspB ∆cspD.*

After 7 dpi, head kidney samples infected with *A. salmonicida* wild type, Δ*cspB*, Δ*cspD*, Δ*cspB*Δ*cspD* were visualized using transmission electron microscopy (TEM). Non-infected lumpfish head kidney tissue, utilized as control, showed a rounded cell morphology with a large nucleus and absence of bacteria (Fig. 6A). Infected lumpfish head kidney with *A. salmonicida* wild type showed similar morphology compared to the non-infected lumpfish head kidney, but intracellular *A. salmonicida* was visualized. In contrast, inflammation, and immune active cells (e.g., vesiculated cells) were observed in the Δ*cspB*, Δ*cspD*, Δ*cspB*Δ*cspD* infected lumpfish head kidney, which relate to the attenuated phenotype of these mutants in lumpfish (Fig. 6B through E).

### Differentially expressed genes

**Fig 7:**
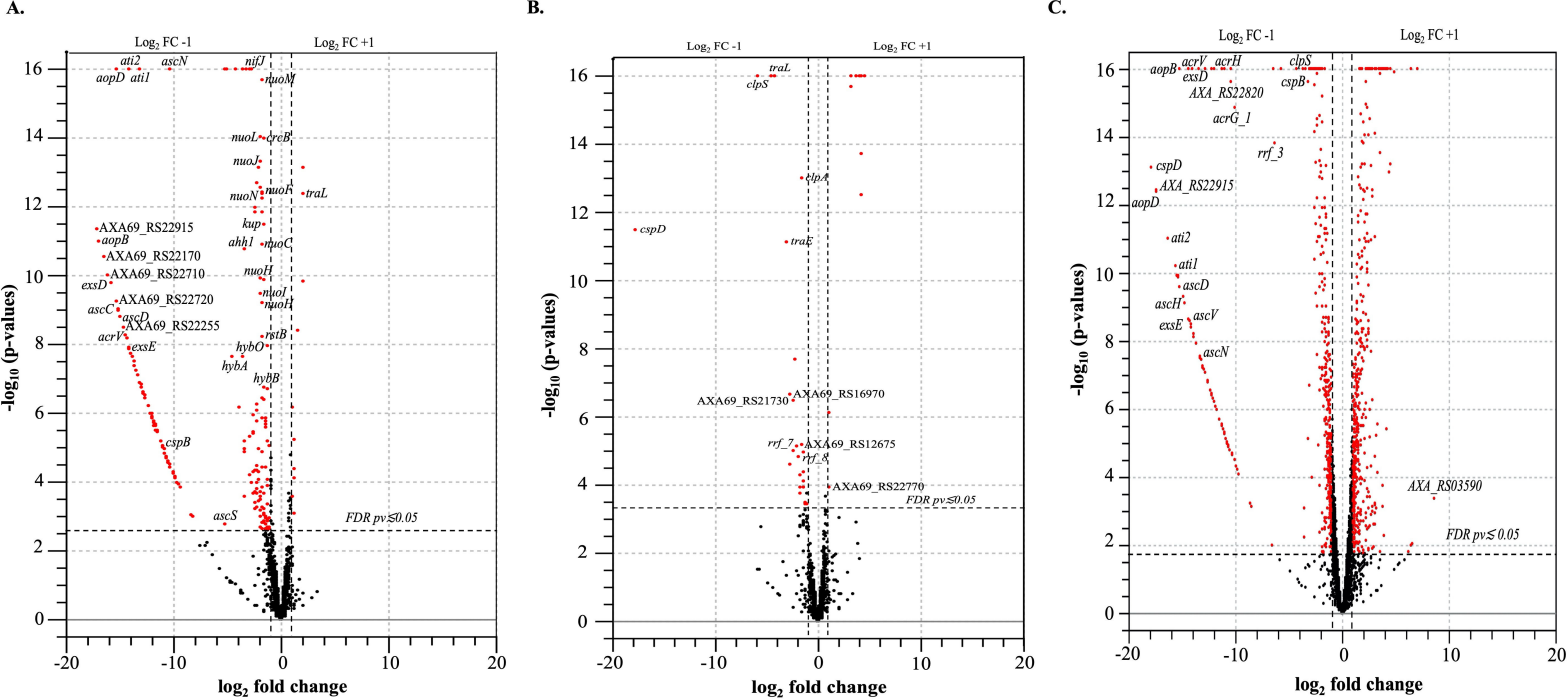
Transcriptomic profiling of ∆*cspB*, ∆*cspD*, and ∆*cspB*∆*cspD* compared to *A. salmonicida* wild type. (A) Volcano plot ∆*cspB* DEGs. (B) Volcano plot of ∆*cspD* DEGs. (C) Volcano plot of ∆*cspB* ∆*cspD* DEGs. Red dots indicate significant DEGs.

**Fig 8:**
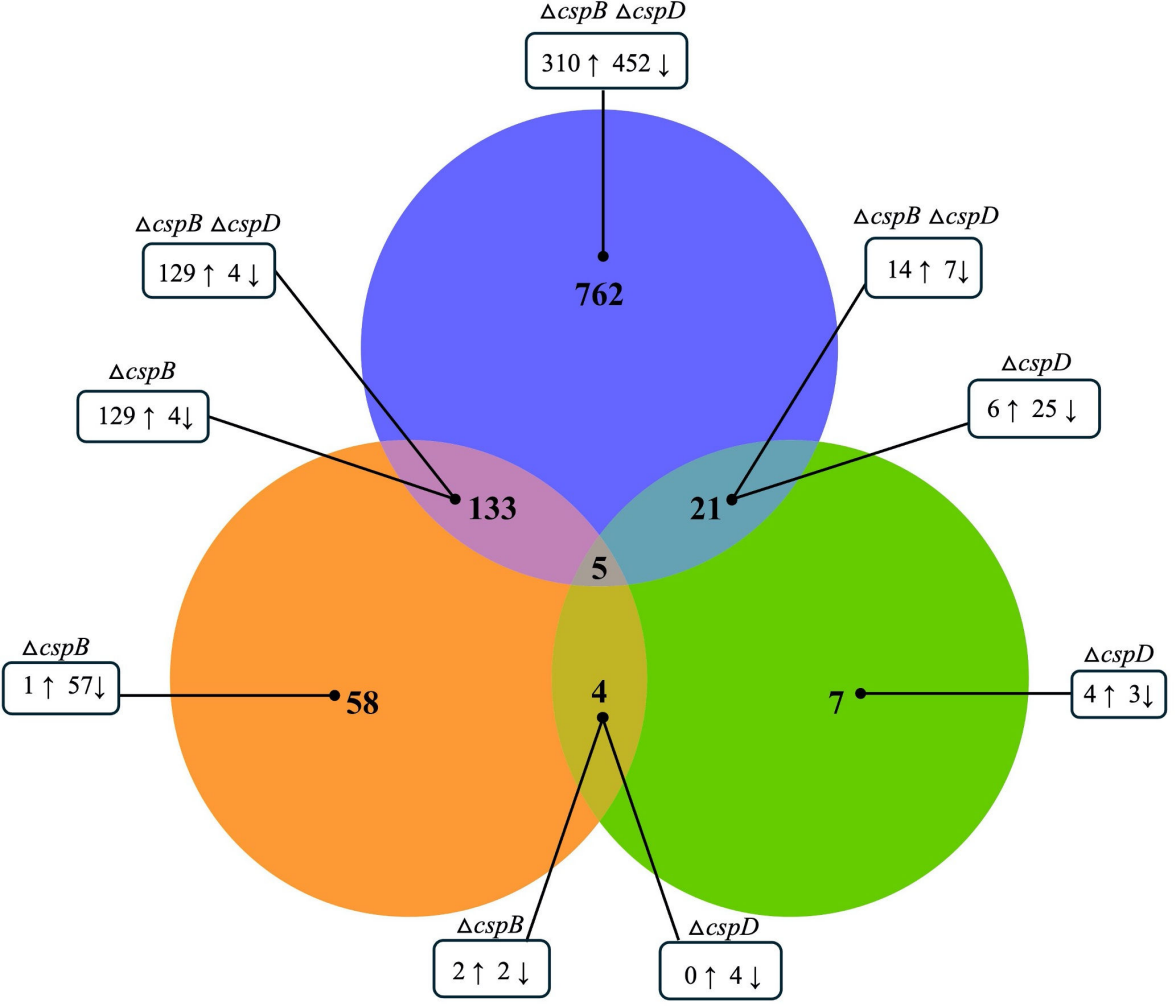
DEG comparison of ∆*cspB*, ∆*cspD*, and ∆*cspB*∆*cspD.* Venn diagram of DEGs.

Cold shock proteins have transcriptional and translational regulation roles, impacting genes related to different processes, including stress resistance, and virulence (90). To get a complete in-depth insight into the function of *A. salmonicida cspB* and *cspD*, a transcriptomics analysis was conducted for Δ*cspB*, Δ*cspD*, and Δ*cspB*Δ*cspD* mutants and compared to the *A. salmonicida* wild type. The total number of raw reads was between 73 and 176 million reads, and the mapped reads were between 96% and 97% (Table S4). Global gene expression indicated strong correlation (*r* ^2^ = 0.9273; *P* ≤ 0.0001 for Δ*cspB*, *r* ^2^ = 0.9797; *P* ≤ 0.0001 for Δ*cspD,* and *r* ^2^ = 0.8424; *P ≤* 0.0001 for Δ*cspB*Δ*cspD*) between control and mutant samples (Fig. S8). Principal component analysis and heat map exhibited a clear separation of control and mutant samples. PC1 explained 46.8% and PC2 explained 20.9% of the total variation in expression data (Fig. S7A through D). Transcriptome analysis revealed that 200 DEGs for Δ*cspB*, 37 DEGs for Δ*cspD*, and 921 DEGs for Δ*cspB*Δ*cspD* were dysregulated according to the defined cutoff criteria (log_2_ FC ≥|1| and FDR *P ≤* 0.05). In *A. salmonicida* Δ*cspB*, 10 genes were upregulated, and 190 genes were downregulated. On the other hand, in *A. salmonicida* Δ*cspD*, 12 genes were upregulated, and 25 genes were downregulated, and for the double mutant Δ*cspB*Δ*cspD*, 473 genes were upregulated, 448 were downregulated. These results indicate that CspB and CspD might act as transcriptional regulators influencing cell division (e.g., *treB*), virulence factors located in the chromosome (e.g., *AXA69_RS07620*, *AXA69_RS22000*, *ahh1*, and *aexT*) and large plasmid (e.g., *exsD *and *aopO*), and ultimately pathogenesis (Supplementary file 1: sheets S1-13, Fig. 7 and 8). The protein-protein interaction showed no direct interaction among CspB or CspD or with other proteins (Fig. S9). These results suggest that CspB and CspD act as transcriptional regulators. In the *A. salmonicida* chromosomes, 109, 37, and 778 genes were differentially regulated in Δ*cspB,* Δ*cspD*, and Δ*cspB*Δ*cspD*, respectively. On the other hand, a total of 92, 6, and 128 genes were differentially regulated in the large plasmid pASal5 for Δ*cspB,* Δ*cspD*, and Δ*cspB*Δ*cspD*. Six genes in plasmid pASal1, six in plasmid pASal2 and three in pASal3 were differentially regulated only for Δ*cspB*Δ*cspD.* Most genes (both in chromosome and large plasmid) for Δ*cspB* were downregulated (Table 2). This result suggested that CspB protein mostly works as a transcriptional repressor.

**TABLE 2:** The number of differentially expressed genes in the chromosome and the plasmids of the mutant strains^*a*^

Strain	Chromosome (NZ_CP048223)	pASal1 (NZ_CP048225)	pASal2 (NZ_CP048226)	pASal3 (NZ_CP048227)	pASal5 (NZ_CP048224)
Δ*cspB*	109 (3 ­↑; 106 ↓)	ND	ND	ND	92 (7 ­↑­; 84 ↓)
Δ*cspD*	37 (10 ­↑­; 21 ↓)	ND	ND	ND	6 (2­ ­↑­; 4 ↓)
Δ*cspB* Δ*cspD*	778 (417 ­­↑; 361 ↓)	6 (5 ­↑; 1 ↓)	6 (6­ ­­↑)	3 (3 ­­↑)	128 (42 ­↑­; 86 ↓)

^*a*^ ND, non-detected; ­↑, upregulated genes; ↓, downregulated genes.

### Gene ontology enrichment analysis

The DEGs in *A. salmonicida* Δ*cspB* were characterized by the enrichment of five GO terms associated with biological process (BP), including oxidoreductase activity, acting on NAD(P)H, quinone or similar compound as acceptor, electron transport chain, cellular respiration, NADH dehydrogenase (quinone) activity, and NADH dehydrogenase (ubiquinone) activity. All differentially expressed genes were associated with only one KEGG pathway related to oxidative phosphorylation. There was no GO term related to cellular components (CCs) and molecular function (MF). Additionally, *nuoN*, a gene related to NADH dehydrogenase I chain N (91), was common in all the biological processes.

**Fig 9:**
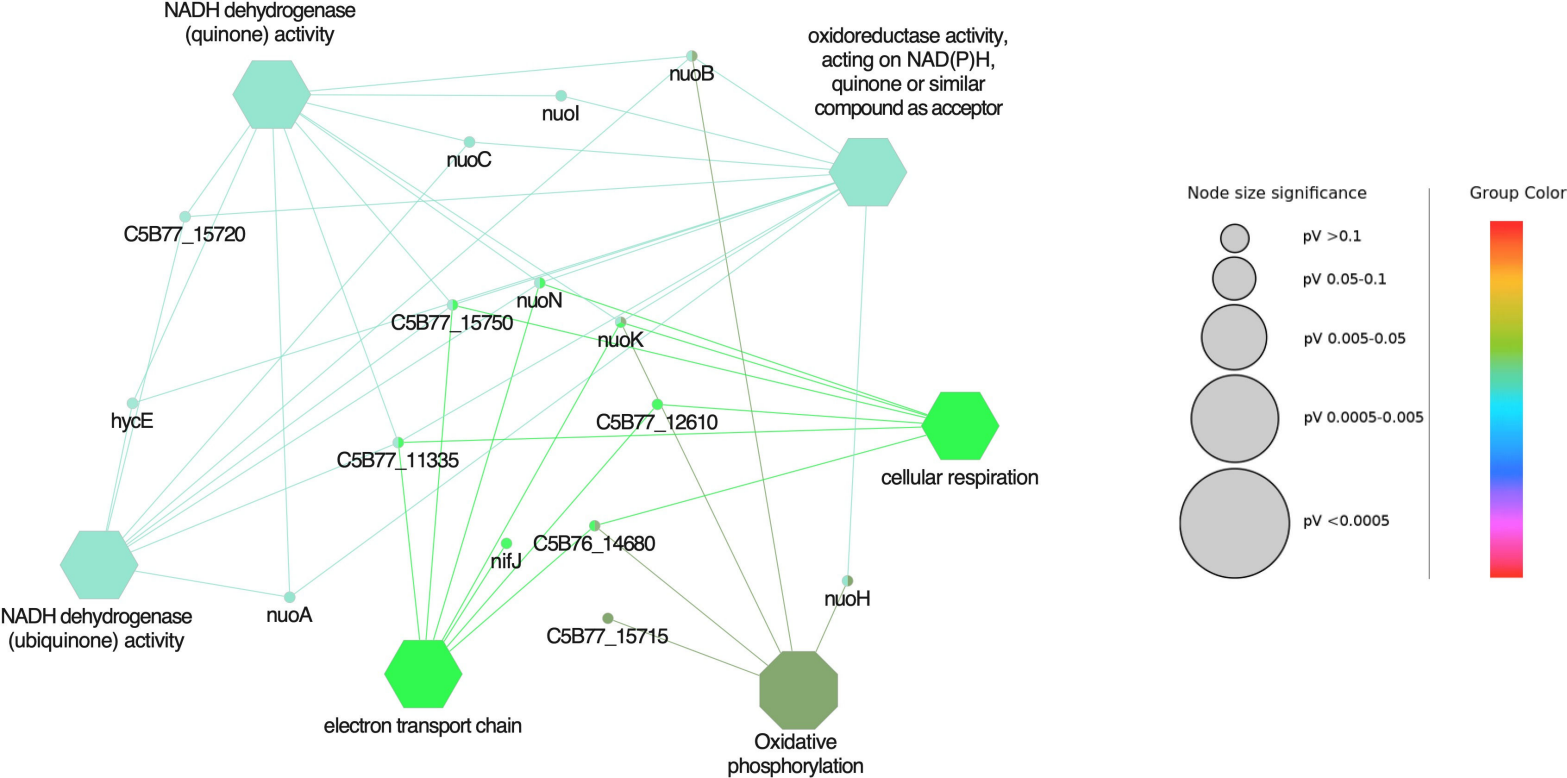
Gene ontology enrichment analysis of DEGs of ∆*cspB.* Functionally grouped the network with terms, where only the label of the most significant term per group is shown. GO categories were separated into biological process, molecular function, and cellular components (Octagon represents KEGG pathway and Hexagon represents biological process) and GO term fusion, medium network specificity, were selected. GO terms and KEGG pathways with a *P* ≤ 0.05 were treated as a significant and Kappa Score Threshold = 0.4 were considered for the analysis. The node size represents the term enrichment significance.

**Fig 10:**
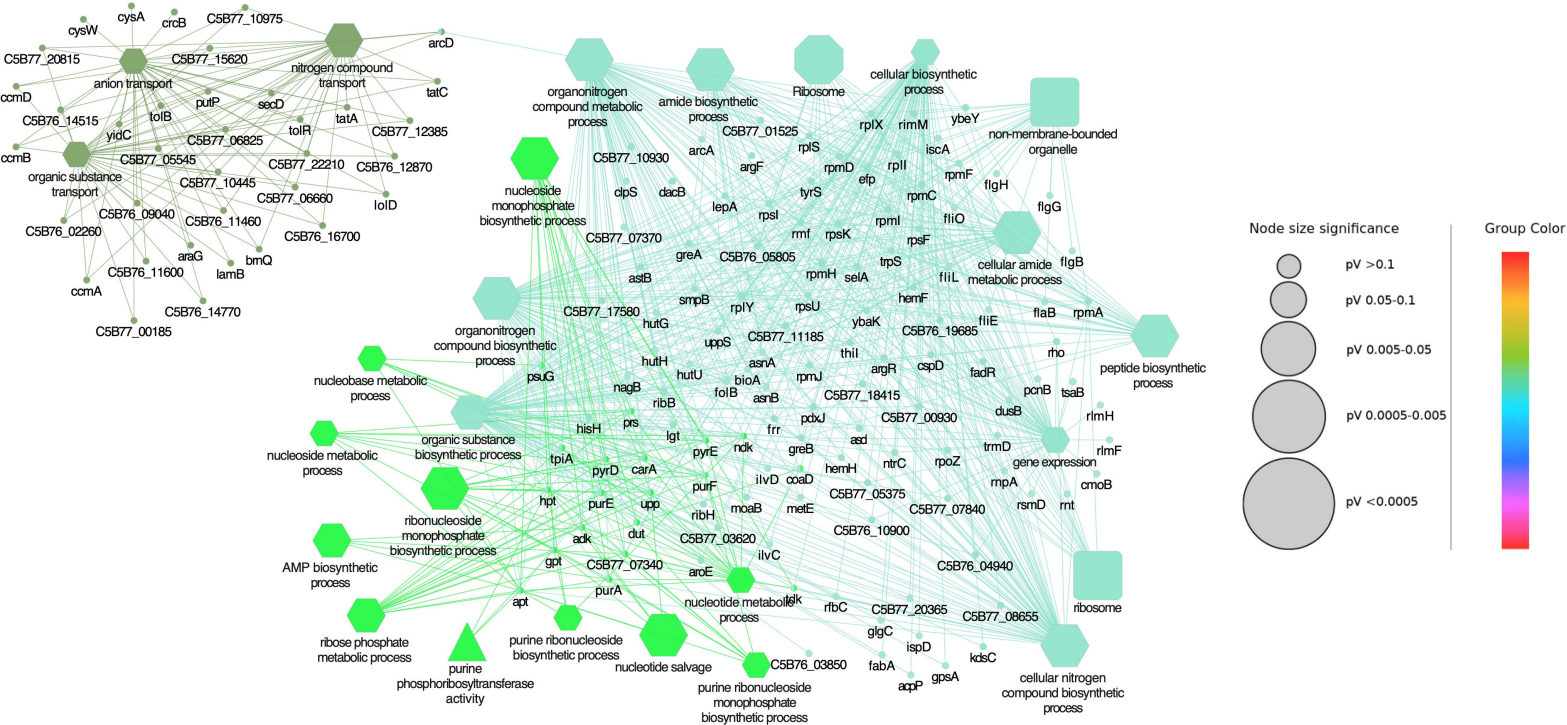
Gene ontology enrichment analysis of DEGs of ∆*cspB* ∆*cspD.* Functionally grouped the network with terms, where only the label of the most significant term per group is shown. GO categories were separated into biological process, molecular function, and cellular components (octagon represents KEGG pathway, hexagon represents biological process, round rectangle represents cellular component, and triangle represents molecular function) and GO term fusion, medium network specificity, were selected. GO terms and KEGG pathways with a *P ≤* 0.05 were treated as a significant and Kappa Score Threshold = 0.4 were considered for the analysis. The node size represents the term enrichment significance.

GO term enrichment analysis showed that the highest number of genes was associated with oxidative phosphorylation, oxidoreductase activity, NADH dehydrogenase (quinone) activity, and NADH dehydrogenase (ubiquinone) activity. In comparison to all other BP, a smaller number of genes were associated with the electron transport chain and cellular respiration pathways, respectively (Supplementary file 2: sheet 1, and Fig. 9). DEGs in *A. salmonicida*Δ*cspB*Δ*cspD* were characterized by the enrichment of 22 GO terms linked with BP (e.g., AMP biosynthetic process, nucleobase metabolic process, gene expression, and cellular biosynthetic process), two GO terms related to CC, including ribosome and non-membrane-bounded organelle and only one GO term connected with MF and KEGG pathway, including purine phosphoribosyl transferase activity and ribosome activity. GO enrichment analysis revealed that the highest number of genes were associated with the organic substance biosynthetic process, cellular biosynthetic process, organonitrogen compound metabolic process, cellular nitrogen compound biosynthetic process, organonitrogen compound biosynthetic process, and gene expression (Supplementary file 2: sheet 2, and Fig. 10).

## DISCUSSION

Csp protein family plays a crucial role in bacterial resilience amidst diverse stressful conditions such as starvation, temperature changes, and virulence (5, 16, 38, 92, 93). This pivotal function of Csp in growth, cold adaptation, and virulence has been documented in mesophilic bacteria, including *Listeria monocytogenes*, *B. melitensis*, *S. enterica, E. fecalis*, and *Xylella fastidiosa* (40 – 43, 94, 95). However, the role of Csp in marine psychrotrophic pathogens, like *A. salmonicida*, has not been explored.

This study sheds light on the previously unexplored domain by elucidating the contributions of DNA-binding proteins CspB and CspD in shaping the physiology and pathogenesis of *A. salmonicida*. The impact of CspB and CspD on growth and survival during stationary phase has been well-documented in other bacterial species (1, 5, 16, 92, 96). However, their specific influence in *A. salmonicida*, especially concerning its adaptation mechanisms in marine environments and pathogenicity, remains a novel and crucial area for investigation. We did not observe growth deficiency in *A. salmonicida* Δ*cspB*, Δ*cspD*, and Δ*cspB* Δ*cspD* at 15°C (Fig. 1A). However, at 28°C, the growth rate of *A. salmonicida* Δ*cspD* was notably slower compared to the wild type, Δ*cspB*, and Δ*cspB* Δ*cspD* mutants (Fig. 1B). These results indicate that CspD plays a crucial role in *A salmonicida* growth at higher temperatures. DNA-binding activities of CspD could affect gene transcription, DNA replication, and DNA repair at high temperatures, which could be helpful for the bacteria to grow or survive at high temperatures (18). Moreover, *A. salmonicida* Δ*cspB* and the double mutant Δ*cspB* Δ*cspD* displayed accelerated growth at 28°C compared to the wild type. Suggesting that CspB might play a role in cellular division processes and its deletion compensates for the effect caused by the deletion of *cspD* in the double mutant. Interestingly, it appears that CspB and CspD are dispensable for normal growth at 15°C for *A. salmonicida*, aligning with previous findings in *B. melitensi* (40), *L. monocytogenes* (1, 97), and *Ralstonia solanacearum* (98). Notably, our results indicated that *A. salmonicida cspD* mutant could survive and grow at warmer temperatures, but with a larger generation time than the growth at optimum temperature. This slower growth might be attributed to protein degradation or reduced metabolic activity in *A. salmonicida* under elevated temperatures (99, 100).

*A. salmonicida* has a large virulence plasmid named pASal5, which plays an important role in virulence (101). *A. salmonicida* chromosome and the large plasmid possess numerous ISs, and a functional Type Three Secretion System (T3SS) located in pASal5, which is essential for its virulence (44, 102, 103). Growth of *A. salmonicida* at temperatures over 24°C triggers endogenous mutagenesis driven by thermal inducible ISs (103), which causes mutation of the gene *vapA* that encodes for the VapA or A-layer. Furthermore, plasmid pAsal5 was shown to get lost at growth temperatures above 20°C, which subsequently affects Type III secretion (TTSS) related genes in *A. salmonicida*. Because TTSS genes in *A. salmonicida* are located on a large thermolabile virulence plasmid (pAsal5) (104). We evaluated the effect of *cspB* and *cspD* mutations on the thermal-induced endogenous mutagenesis by quantifying the frequency of VapA mutation under heat stress. We determined that deletions of *cspB* and *cspD* did not influence the frequency of endogenous (Fig. 1C). However, *A. salmonicida* ΔcspD showed a delayed increase in A^−^ colonies under heat stress. This delay in the frequency of endogenous mutagenesis is likely caused by the slower growth displayed by *A. salmonicida* ΔcspD (Fig. 1B). These results suggest that endogenous mutagenesis is growth dependent and CspB and CspD do not influence ISs activity. So, for *A. salmonicida*, it is necessary to maintain a growth temperature lower than 20–22°C to keep the large virulence plasmid pAsal5 with the TTSS-related genes intact, since temperatures over 22°C will cause endogenous mutagenesis of the TTSS-related genes, in turn, reducing virulence (44, 104, 105).

Also, we evaluated the role of *cspB* and *cspD* genes in *A. salmonicida* survival under 0°C and 4°C in TSB and seawater (Fig. 2A through D). We found that Δ*cspD* was more susceptible to low temperatures than the wild type and other mutants. It seems that CspD is more relevant for *A. salmonicida* adaption to the cold than CspB. Previous studies also found that cold shock proteins are vital in bacterial growth at low temperatures (1, 5, 106 – 108). For example, deletion of *cspD* in *L. monocytogenes* and *cspB* in *Caulobacter crescentu* showed a negative effect on survival at low temperatures (4°C and 10°C) (1, 109). Also, it was shown that increased synthesis of cold shock proteins was necessary to acclimate *Bacillus subtilis* to cold temperatures (15°C) (92).

Bile salts are water-soluble end products of hepatic cholesterol released in the small intestine to solubilize lipids (110). Bile salts play an anti-bacterial role in the gut mucus and are involved in fish anti-bacterial innate immunity (111, 112). Bile causes multifaceted deleterious impacts on microbes, and oral-gastric bacteria must overcome bile salt antimicrobial activity (113). Cold shock proteins of bacteria influence bile salt resistance (114). From our experiments, we have found that *A. salmonicida* Δ*cspB*Δ*cspD* mutant was susceptible to bile salts in contrast to the wild type and the single mutants (Fig. S5). Our results also suggested that both, CspB and CspD play a role in resistance to bile salts, perhaps by modulating genes related to membrane synthesis (115). It was shown that in *S. enterica*, both CspC and CspE are essential to resist bile salt stress. In contrast, single *S. enterica* mutants of *cspC* or *cspE* did not show any growth defect in the presence of bile (41). Previous studies found that LPS and OMP contribute to bile resistance, and synthesis of LPS and OMP are also directly linked to the cold shock proteins. Deleting cold shock protein could affect LPS and OMP synthesis, further hampering a pathogen’s bile salt resistance process (115, 116). We evaluated the LPS and OMP profiles of *A. salmonicida cspB* and *cspD* mutants. Though survival was unaffected by the presence of bile salt, disruption in both LPS and OMP was noticed only in Δ*cspD* (Fig. 3A and C). These results suggested that *cspD* plays an essential role in maintaining cell membrane integrity. Similarly, it was reported that CspE significantly contributed to bile resistance in *S*. Typhimurium and was also needed to maintain OMP integrity. However, deleting *cspE* did not impact the LPS profile of *S.* Typhimurium (115, 117).

Biofilm assays showed that *cspD* plays a role in *A. salmonicida* biofilm formation. Confocal microscopy observations showed that *A. salmonicida* Δ*cspD* mutant gained the ability to form a strong biofilm. Δ*cspB* and Δ*cspB*Δ*cspD*, and wild-type strains, did not produce biofilm (Fig. 4). It is possible that membrane modification in *A. salmonicida* Δ*cspD* is related biofilm formation. It is also possible that *cspD* gene could negatively regulate biofilm-related gene expressions in *A. salmonicida*. It was observed that *cspD* in *E. coli* was involved in biofilm formation (118, 119). Liu et al. (98) noticed that *cspD* was not necessary for the biofilm formation of *R. solanacearum* (98). Also, CspC and CspE, CspA, CspV have been found necessary for biofilm formation in *S. enterica*, *Acinetobacter baumannii*, *S. aureus*, and *Vibrio cholera* (41, 88, 120, 121).

Deletion of Csp encoding genes can lead to the bacteria attenuation (41, 42, 88, 89). Our study found that the CspB and CspD also regulate virulence in *A. salmonicida*. For instance, *A. salmonicida* mutants were not able to establish a systemic infection, where *A. salmonicida* Δ*cspB* and Δ*cspD* single mutants were attenuated and Δ*cspB*Δ*cspD* was non-lethal in a lumpfish, while the wild type causes 100% mortality (Fig. 5A). The attenuation facilitated an impaired capability to survive and colonize in lumpfish tissues (Fig. 5D and E). Transmission electron microscopic results showed that lumpfish head kidney cells infected with *A. salmonicida* mutants exhibit higher immune activity compared to wild type and non-infected fish head kidney cells. Also, no bacterial cells were noticed in head kidney cells infected with Δ*cspB*Δ*cspD* mutant (Fig. 6E). It was previously described that double deletion of *csp* genes in *S. enterica* Serovar Typhimurium caused fully attenuation in mice (41), and deletion of all the cold shock proteins caused full attenuation in *L. monocytogenes* in zebrafish (*Danio rerio*) infection model (122). Similar results were observed after deletion of *cspB* and *cspD* in *L. monocytogenes*, where the infection was significantly reduced and became susceptible to stressful conditions and lost its ability to proliferate within host cells (123, 124). It was also noticed that a single deletion of the *cspR and cspD3* gene in *Enterococcus faecalis* and *R. solanacearum* caused attenuation in insects (*G. mellonella*) and tobacco plant hosts, respectively (42, 98).

**Fig 11:**
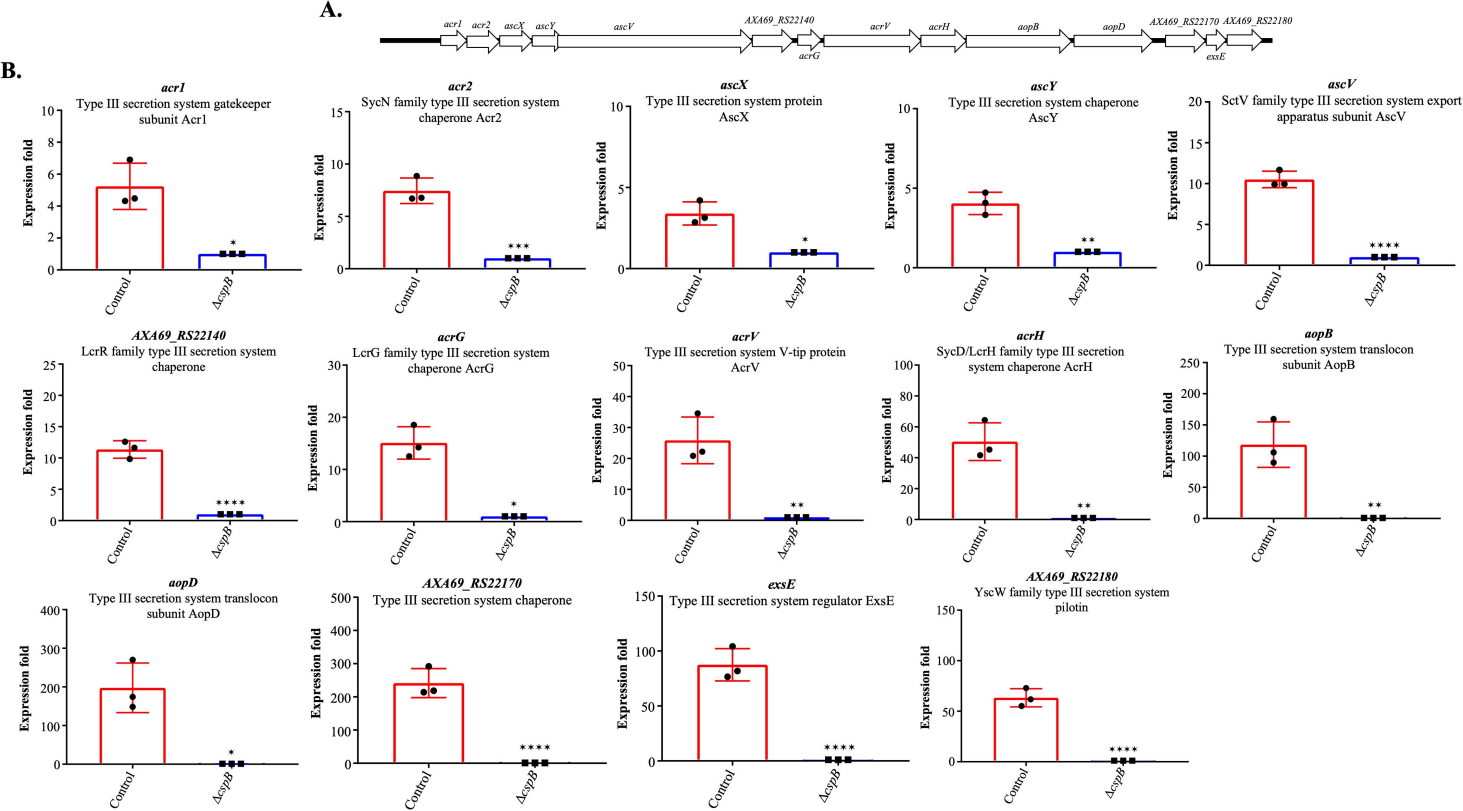
Transcripts per million values (TPMs) plots of T3SS located in pASal5 in *A. salmonicida* Δ*cspB* grown in 15°C. (A) Genetic map of T3SS-related genes located in pASal5. (B) TPM expression folds of significant 14 DEGs in ∆*cspB* mutant vs wild type (*P ≤ 0.05, **P ≤ 0.01, ***P ≤ 0.001, ****P ≤ 0.0001, the nonparametric Kruskal-Wallis test, followed by Dunn’s multiple *post hoc* test, was done to determine the significant differences).

Under optimal growth conditions, TSB at 15°C, significant number of deferentially expressed genes were observed for *A. salmonicida* Δ*cspB*, Δ*cspD*, and double mutant Δ*cspB*Δ*cspD.* Virulence genes, located in the large virulence plasmid pASal5, were downregulated in Δ*cspB* and Δ*cspB*Δ*cspD*, including T3SS related genes (e.g., *aopB*, *acrV*, and *ati2*) (Fig. 7A and C, 11A and B; Fig. S14A and B). These results indicate that CspB positively regulates gene expression of pASal5 virulence plasmid. However, we did not identify Csp binding sites in the promoter regions in the operons of pAsal5, suggesting that CspB might be indirectly participating in the transcriptional regulation of virulence genes in the plasmid. Deletion of *cspD* gene did not affect the expression of any type III secretion-related genes (Fig. 7B; Fig. S12A and B). Similarly, it was found that all three *csp* genes present in *L. monocytogenes* did not have the same role in virulence regulation. *L. monocytogenes cspB* and *cspD* have a critical contribution to virulence, whereas the *cspA* gene was most crucial in stress response rather than virulence (122).

*A. salmonicida cspD* seems more related to stress response than virulence (Fig. S10A and B, S11A and B, S13A and B). For instance, in *A. salmonicida* Δ*cspD* mutant, most of the ribosomal protein-related genes (16S ribosomal RNA, 23S ribosomal RNA, 5S ribosomal RNA) were downregulated, which indicated disturbance in cellular homeostasis (125). Also, downregulation of AAA family ATPase protein gene could hampers diverse cellular activities (126). Downregulation of ATP-dependent Clp protease ATP-binding subunit ClpA indirectly suggests impaired normal growth and development (127 – 129), which agrees with the slow growth observed in *A. salmonicida* Δ*cspD* mutant at 28°C (Fig. 1B). Downregulation of *tra* (conjugal transfer system) genes possibly reflects the trouble in exporting macromolecules, including virulence factors (130). Altogether, these observations revealed that deletion of *cspD* gene regulates critical cellular components and increases structural damage to the DNA, cell wall, and outer membrane, further affecting bacterial growth, survival, and virulence. Previously, from transcriptomic studies of *B. melitensis* and *S. enterica*, it was also found that Csp extensively regulate various genes, including those linked to virulence (41, 89).

Additionally, protein-protein interaction analysis showed that CspB and CspD were not directly linked to other proteins, which indicates that these two proteins could function as transcriptional/translational regulators (Fig. S9). Early studies also found that in *T. thermophilus* Csp were functioning as translational regulators (45).

Complementation for *A. salmonicida* was not possible. It was because *A. salmonicida* has four plasmids, including large plasmids that did not allow inserting a new plasmid in it. However, further studies are required to know the exact immunostimulatory mechanisms and gene expression regulatory mechanisms of Csp of *A. salmonicida.*

### Conclusion

Our study is the first attempt to elucidate how the cold shock proteins of *A. salmonicida* impact its ability to withstand stresses, including low temperature, bile-induced stress, and virulence. We noticed that CspD plays a major role in the *A. salmonicida* growth, as deletion of *cspD* delay growth and growth dependent-endogenous mutagenesis (Fig. 1B and C). Deletion of *cspD* gene induced biofilm formation in *A. salmonicida*, suggesting that CspD represses biofilm synthesis (Fig. 4A through D). Also, we found that *cspD* deletion affects LPS synthesis (Fig. 3A), which seems related to biofilm formation. *A. salmonicida* double mutant Δ*cspB*Δ*cspD* seems to compensate for the Δ*cspD* phenotype. *A. salmonicida* Δ*cspB*Δ*cspD* showed a smooth LPS profile and did not produce biofilm. These results suggest that CspB might play a role in the biofilm suppression.

Both *cspB* and *cspD* affect *A. salmonicida* virulence in lumpfish, and Δ*cspB*Δ*cspD* was fully attenuated (Fig. 5A). Transcriptomics analysis revealed that *cspB* regulates more genes related to physiology and virulence than *cspD*. Also, deletion of *cspD* did not affect virulence-related genes in the large plasmid pASal5. Around 778 genes in the chromosome and 143 genes in the plasmids were dysregulated in the double Δ*cspB*Δ*cspD* mutant. These results indicate that both genes together control the expression of many genes related to various physiological and virological functions of *A. salmonicida*. Overall, both DNA-binding cold shock proteins have an essential role in controlling differential gene expressions, which further affects *A. salmonicida* physiology, and virulence.

## Data Availability

The raw data were deposited in the NCBI BioProject database under the accession number PRJNA982552.

## SUPPLEMENTAL MATERIAL

Supplemental figures iai.00011-24-s0001.pdf Figures S1 to S15.

Supplemental file 1 iai.00011-24-s0002.xlsx Differentially expressed genes (DEGs) of mutants by reference genome-guided transcriptome assembly (log2FC{greater than or equal to}1, FDR{less than or equal to}0.05).

Supplemental file 2 iai.00011-24-s0003.xlsx GO analysis.

Supplemental tables iai.00011-24-s0004.docx Tables S1 to S4.

ASM does not own the copyrights to Supplemental Material that may be linked to, or accessed through, an article. The authors have granted ASM a non-exclusive, world-wide license to publish the Supplemental Material files. Please contact the corresponding author directly for reuse.
