# Supplementary material for: Role of cold shock proteins B and D in Aeromonas salmonicida subsp. salmonicida physiology and virulence in lumpfish (Cyclopterus lumpus)
Source: Infect Immun. 2024 Jun 26;92(8):e00011-24. doi: 10.1128/iai.00011-24 (PMC11320987; doi:10.1128/iai.00011-24)
Supplement: Supplemental figures — Figures S1 to S15. [file iai.00011-24-s0001.pdf]

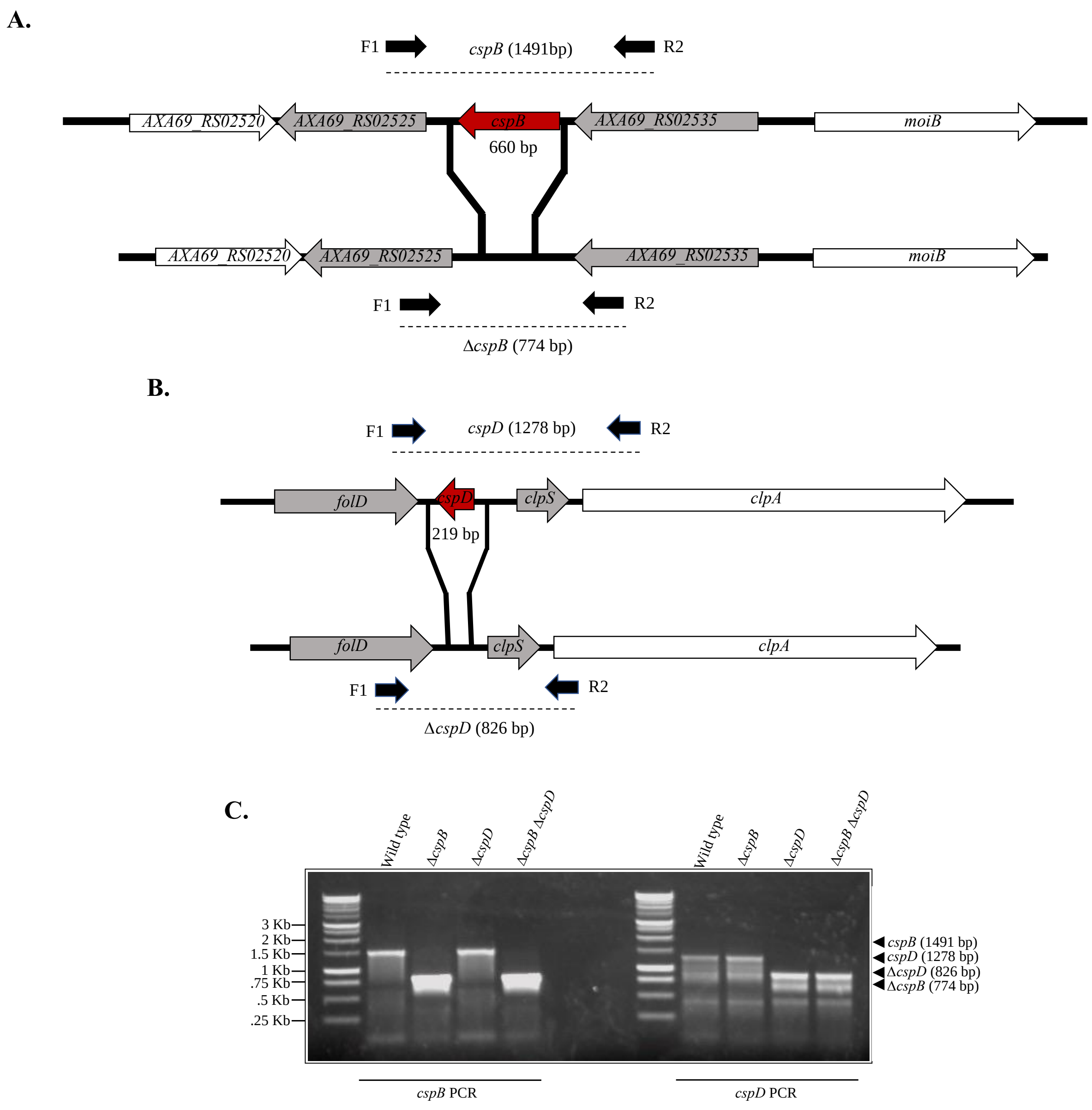

Figure S1. ***A. salmonicida csp* mutant construction. A.** *cspB* genetic map; **B.** *cspD* genetic map; **C.** PCR mutant verification.

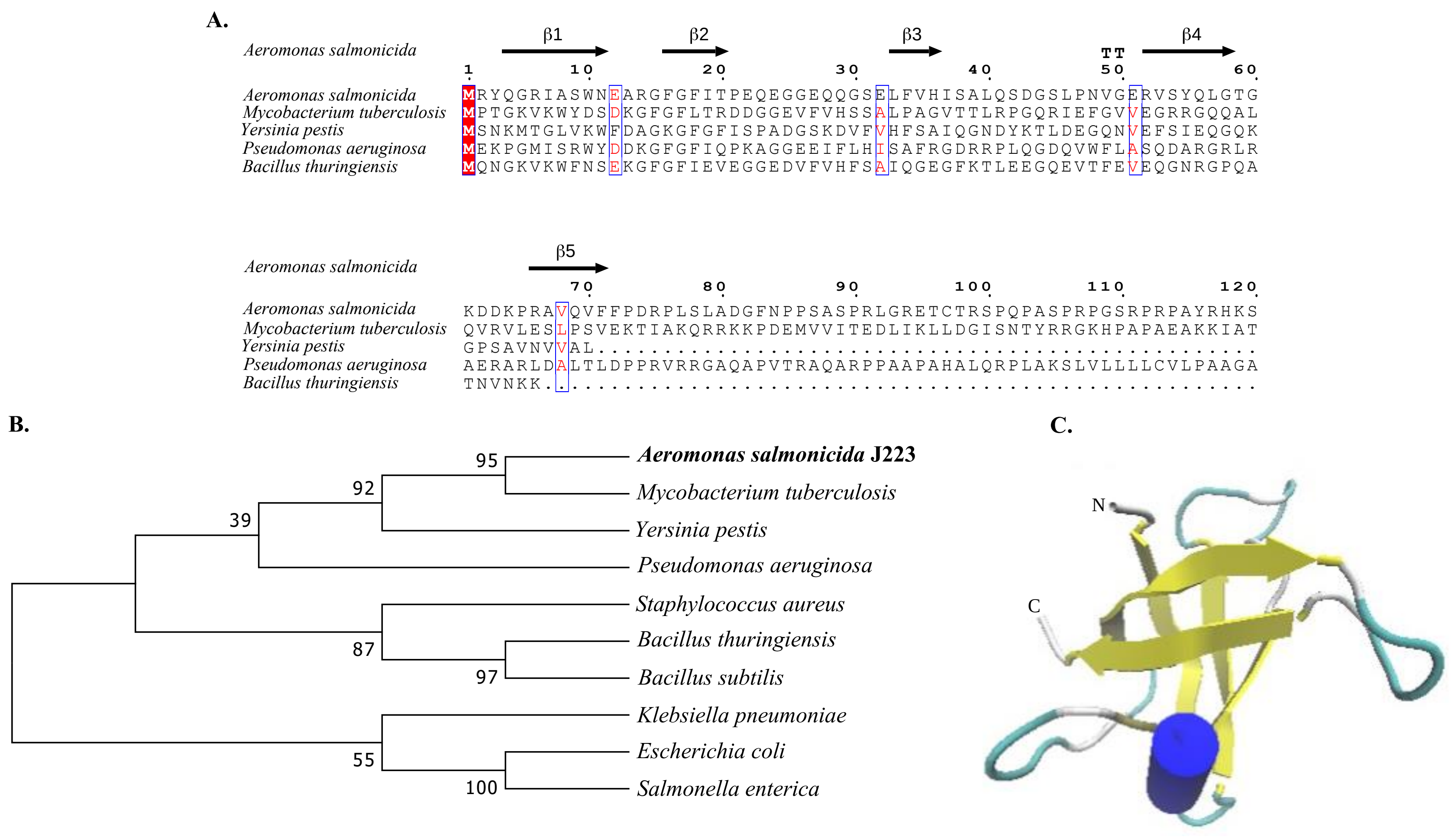

Figure S2. Alignments, 3D, and mutant. A. Amino acids comparison of CspB from different organisms. The secondary structures at the top and bottom of the alignment corresponds to the *A. salmonicida* CspB and *B. thuringiensis* CspB, respectively (arrows represent β-sheet); **B.** Phylogenetic tree of CspB. The evolutionary history was inferred using the Neighbor-Joining method. The optimal tree with the sum of branch length = 2.06810462 is shown. The percentage of replicate trees in which the associated taxa clustered together in the bootstrap test (1000 replicates) are shown next to the branches. The tree is drawn to scale, with branch lengths in the same units as those of the evolutionary distances used to infer the phylogenetic tree. The evolutionary distances were computed using the p-distance method and are in the units of the number of base differences per site. The analysis involved 10 nucleotide sequences. All positions containing gaps and missing data were eliminated. There was a total of 184 positions in the final dataset. Evolutionary analyses were conducted in MEGA7; **C.** Protein Structure of CspB.

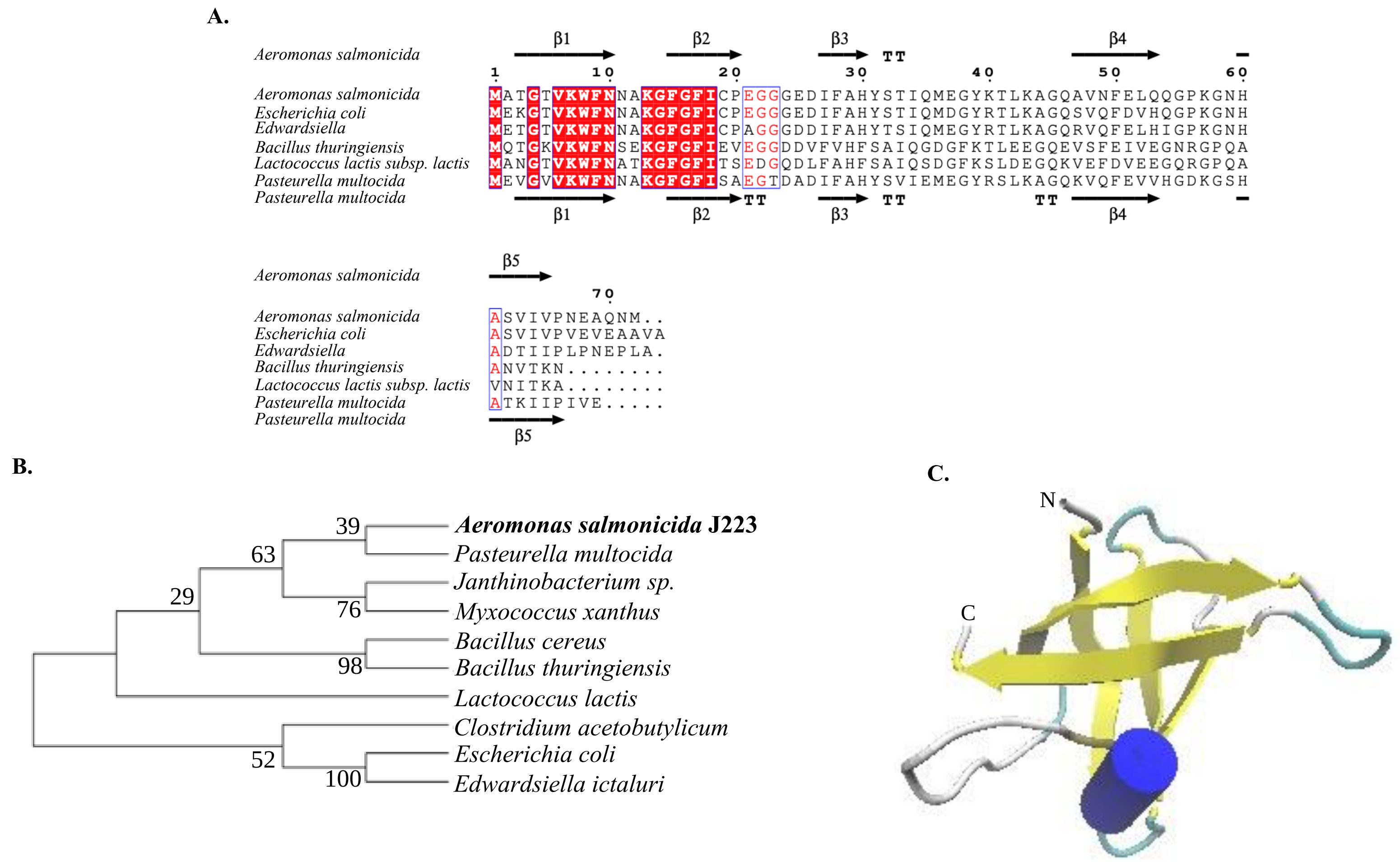

Figure S3. Alignments, 3D, and mutant. A. Amino acids comparison of CspD from different organisms. The secondary structures at the top and bottom of the alignment corresponds to the *A. salmonicida* CspD and *P. multocida* CspD, respectively (arrows represent β-sheet); **B.** Phylogenetic tree of CspD. The evolutionary history was inferred using the Neighbor-Joining method. The optimal tree with the sum of branch length = 2.06810462 is shown. The percentage of replicate trees in which the associated taxa clustered together in the bootstrap test (1000 replicates) are shown next to the branches. The tree is drawn to scale, with branch lengths in the same units as those of the evolutionary distances used to infer the phylogenetic tree. The evolutionary distances were computed using the p-distance method and are in the units of the number of base differences per site. The analysis involved 10 nucleotide sequences. All positions containing gaps and missing data were eliminated. There was a total of 184 positions in the final dataset. Evolutionary analyses were conducted in MEGA7; **C.** Protein Structure of CspD.

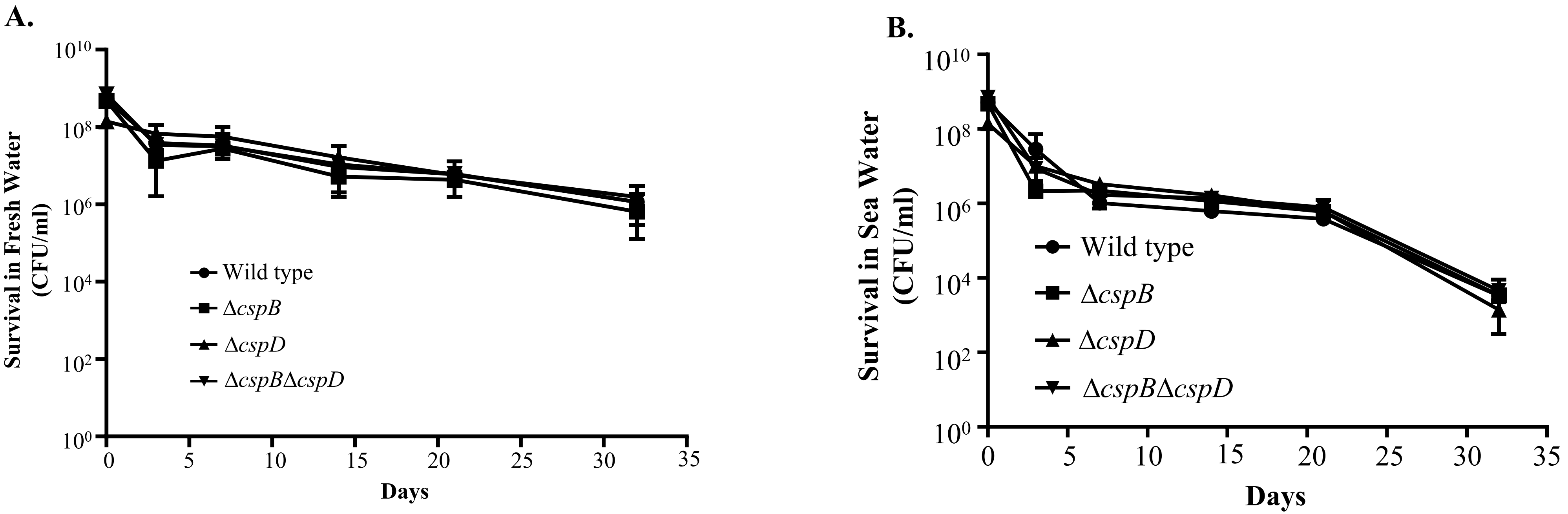

Figure S4. *A. salmonicida* survival in fresh and sea water. *A. salmonicida* wild type, mutants were incubated in fresh water and sea water for 32 days. **A.** Survival rates of wild Type, Δ*cspB*, Δ*cspD*, Δ*cspB* Δ*cspD* in fresh water; **B**. Survival rates of wild Type, Δ*cspB*, Δ*cspD*, Δ*cspB* Δ*cspD* in sea water.

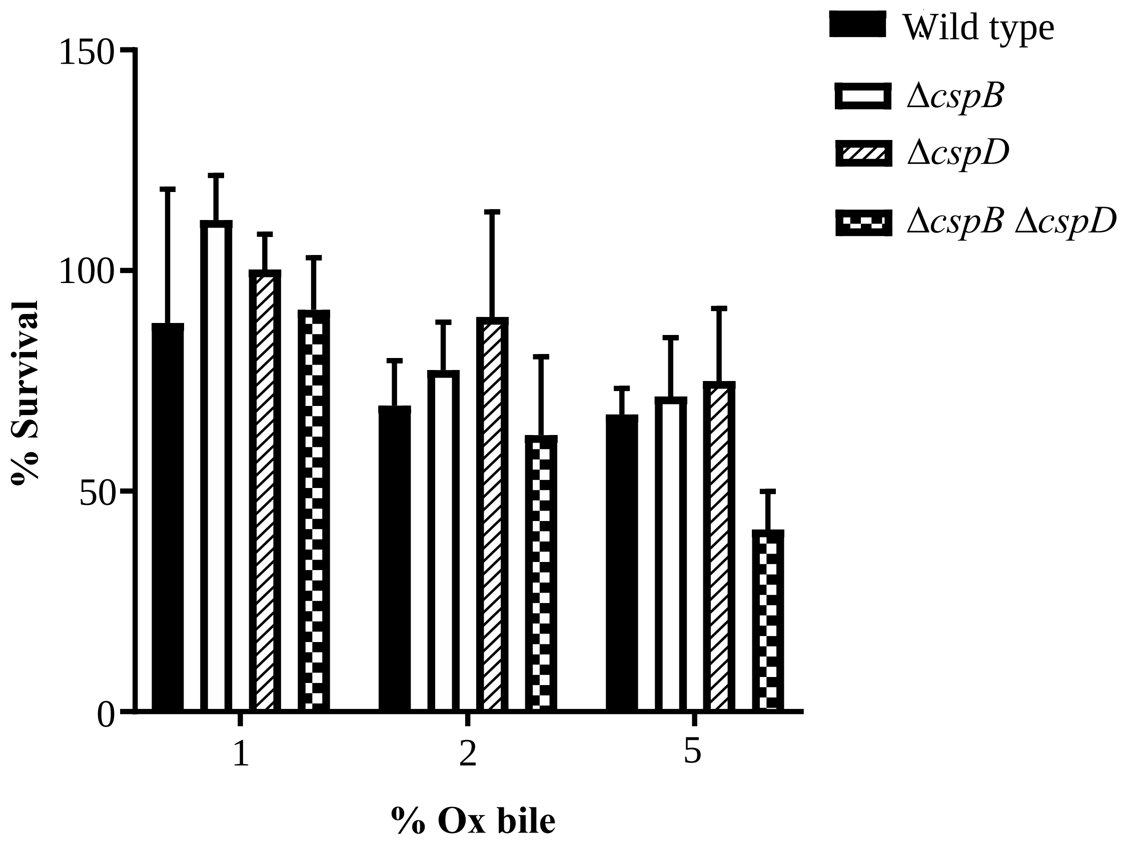

Figure S5. ***A. salmonicida*** **Ox bile tolerance.** *A. salmonicida* wild type and mutants (3 biological replicas for each strain) survival in Ox bile containing agar plates (Ox bile concentrations were 1, 2, and 5%). Survival rates were not significantly different between mutants and the wild type of strain ($p \leq 0.05$).

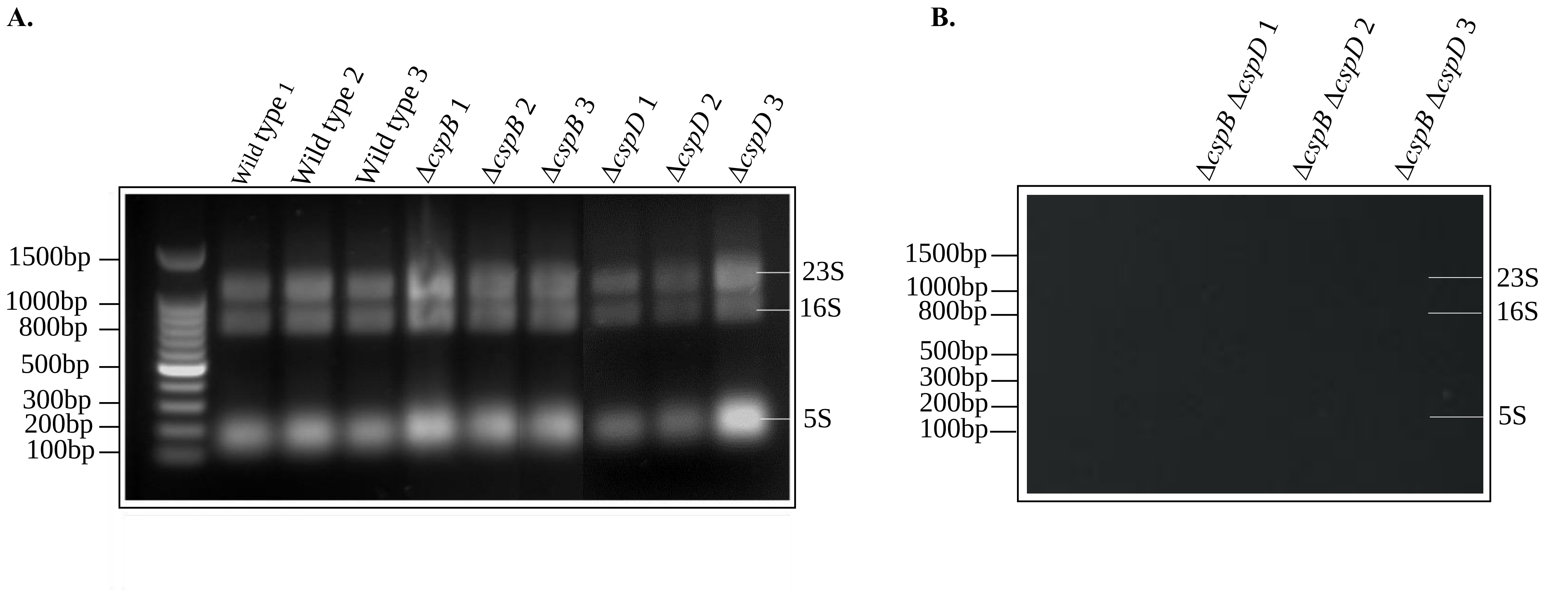

Figure S6. RNA integrity in agarose gel 1%. A. RNA of *A. salmonicida* wild type, Δ*cspB*, Δ*cspD*; **B.** RNA of *A. salmonicida* Δ*cspB* Δ*cspD*.

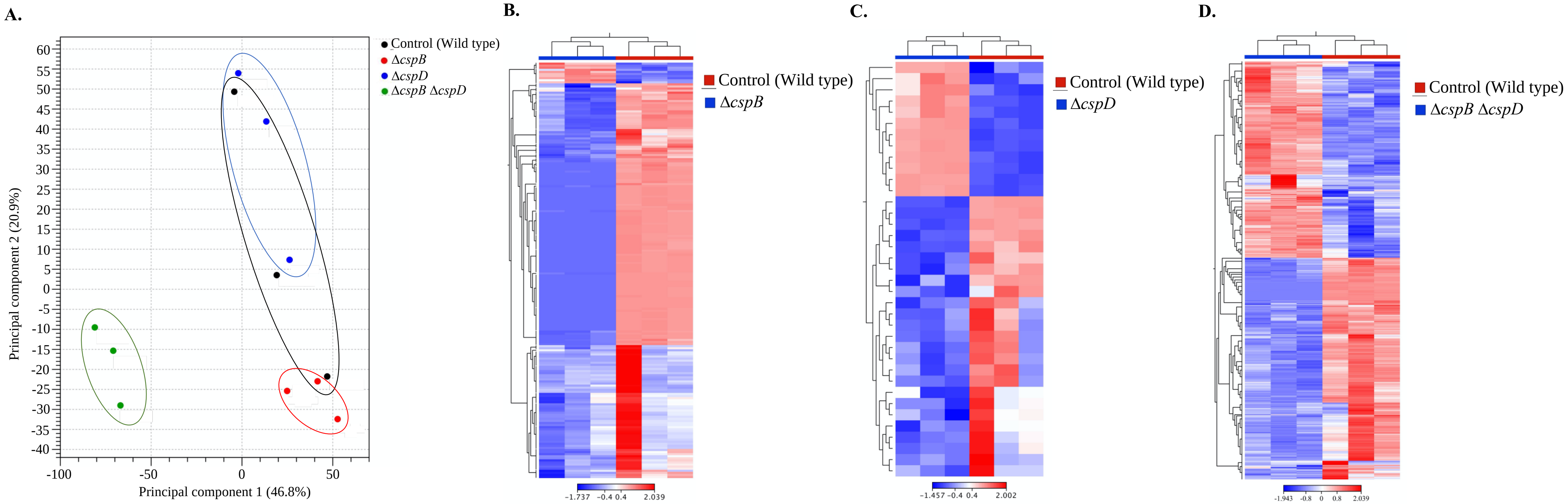

Figure S7. Effect of deletion of *cspB* and *cspD* in *A. salmonicida* global transcriptomic response in TSB at 15 °C. *A. salmonicida* strain were grown in TSB at 15 °C until O.D. 0.7. A total of 12 RNA libraries comprising 3 biological replicates for 4 different strains (wild type, Δ*cspB*, Δ*cspD* and Δ*cspB* Δ*cspD*) were analyzed. **A.** Principal component analysis (PCA) of control (wild type) and mutant strains Δ*cspB*, Δ*cspD* and Δ*cspB* Δ*cspD*, based on TPMs; **B.** Heat map of Δ*cspB* vs wild type; **C.** Heat map of Δ*cspD* vs wild type; **D.** Heat map of Δ*cspB* Δ*cspD*.

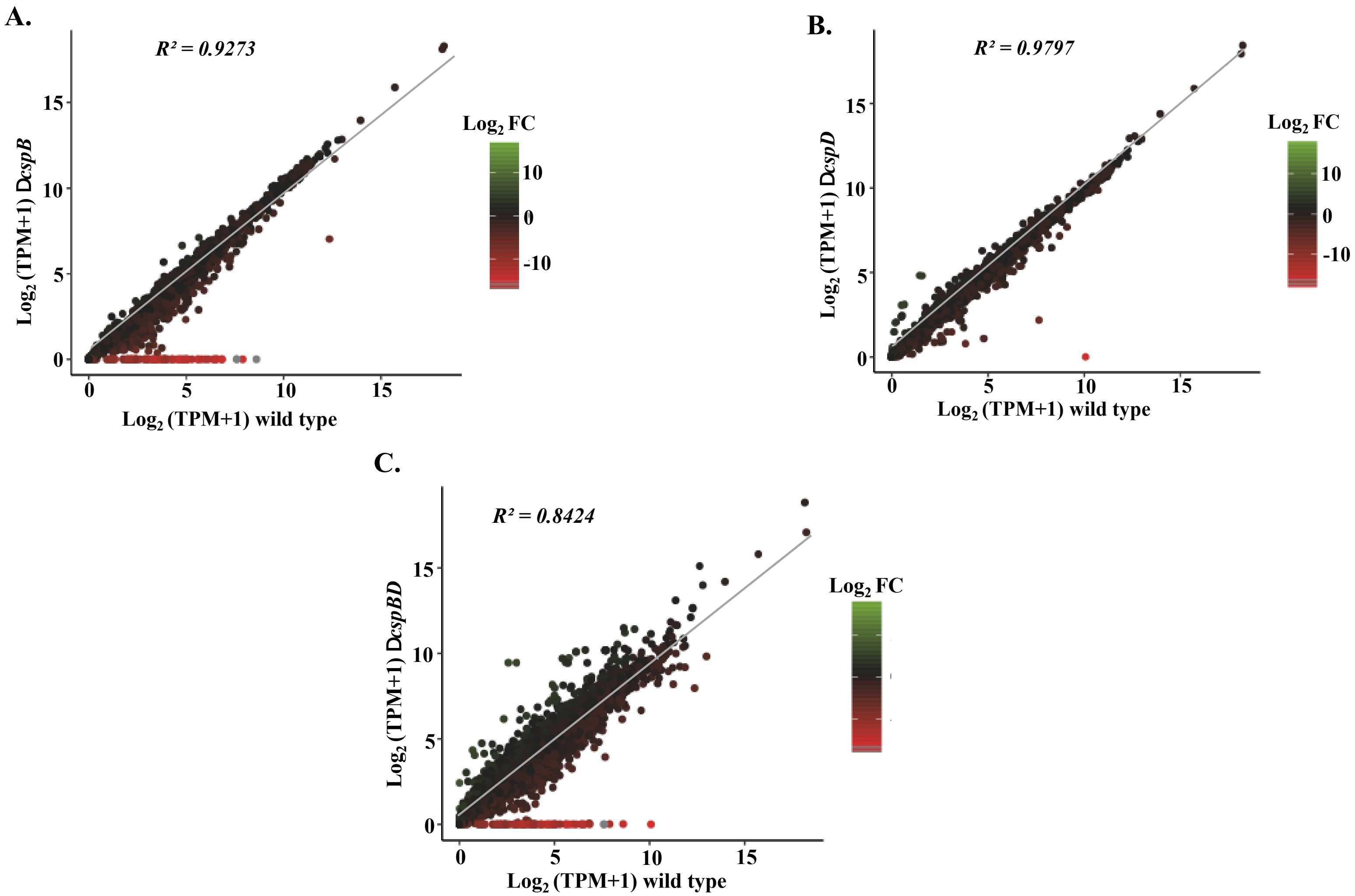

Figure S8. Global gene expression. **A.** Scatter plot of RNA-seq expression of control (wild type) and Δ*cspB*; **B.** Scatter plot of RNA-seq expression of control (wild type) and Δ*cspD*; **C.** Scatter plot of RNA-seq expression of control (wild type) and Δ*cspB* Δ*cspD*. Each dot represents a gene where red, green and represent down-, up-regulated and non-differentially expressed genes, respectively.

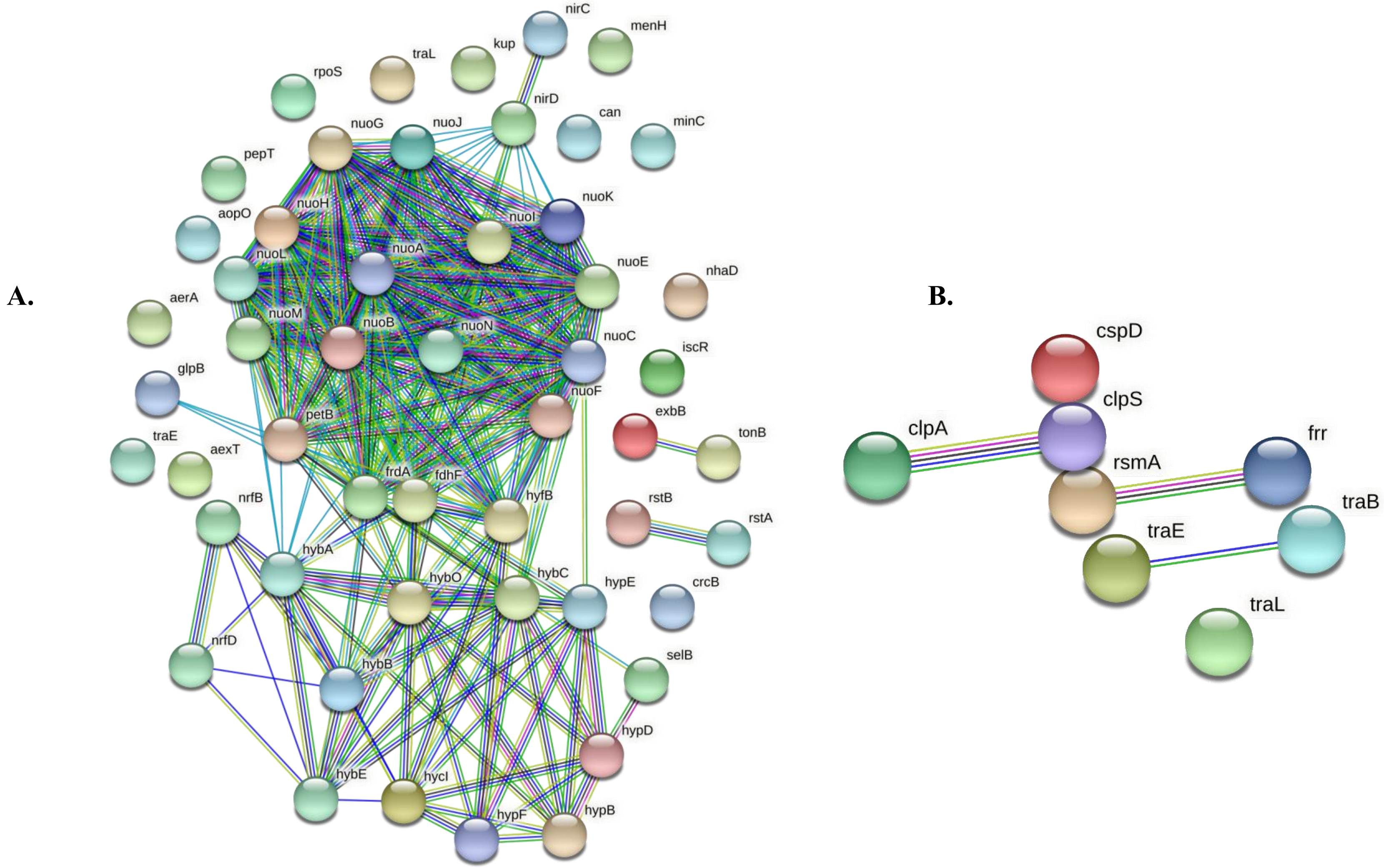

Figure S9. Protein-protein interaction. A. Protein-protein interaction of CspB; **B.** Protein-protein interaction of CspD. colored nodes: query proteins and first shell of interactors; white nodes: second shell of interactors; empty nodes: proteins of unknown 3D structure. Filled nodes: some 3D structure is known or predicted.

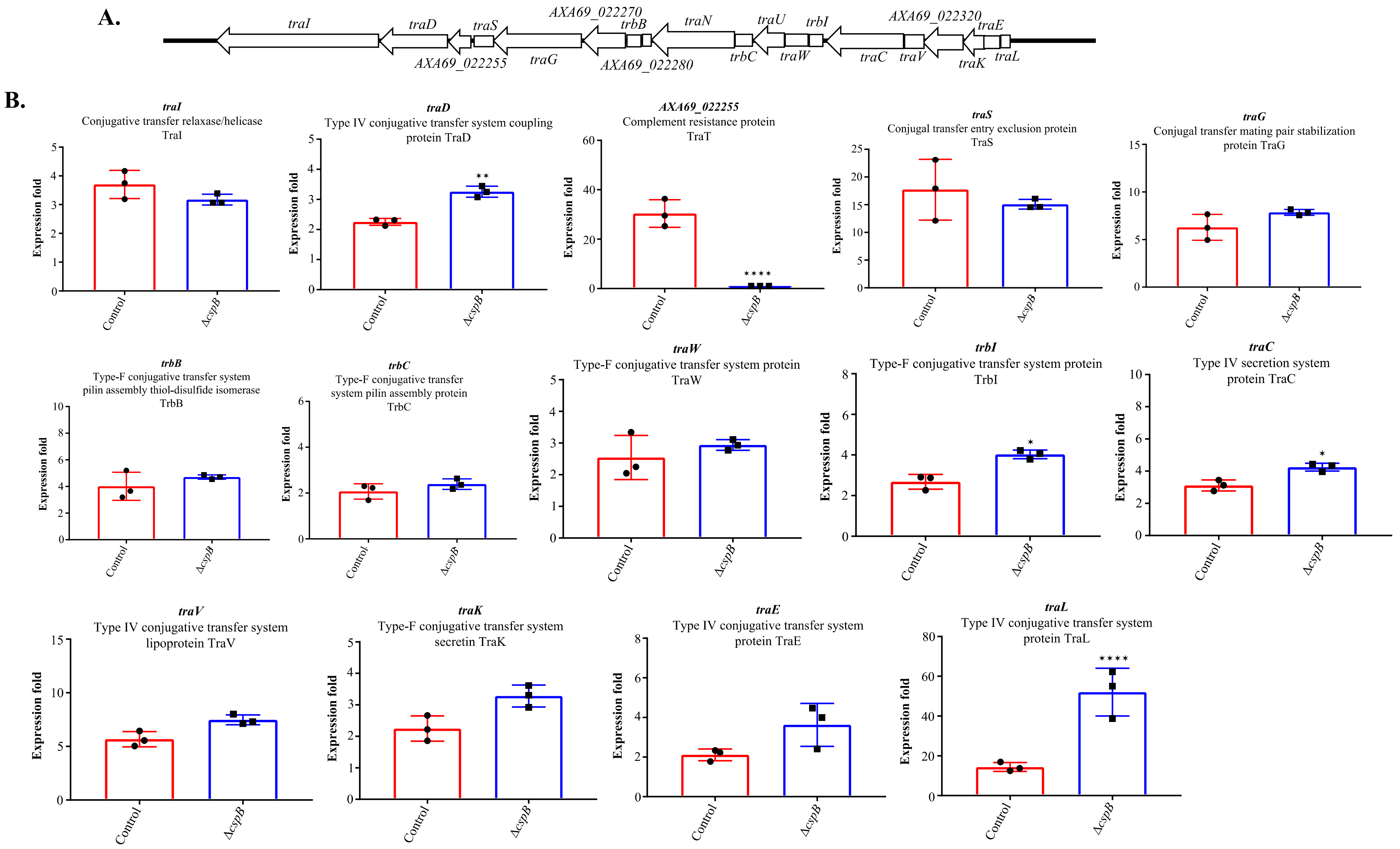

Figure S10. Transcripts per million values (TPMs) plots of transfer genes (*tra* operon) located in pASal5 in *A. salmonicida* Δ*cspB* grown in 15 °C. A. Genetic map of transfer related genes located in pASal5; **B.** TPMs expression folds of significant 14 DEGs in Δ*cspB* mutant vs wild type (*$p \leq 0.05$, **$p \leq 0.01$, ***$p \leq 0.001$, ****$p \leq 0.0001$, The non-parametric Kruskal-Wallis test, followed by Dunn's multiple post-hoc test, was done to determine the significant differences).

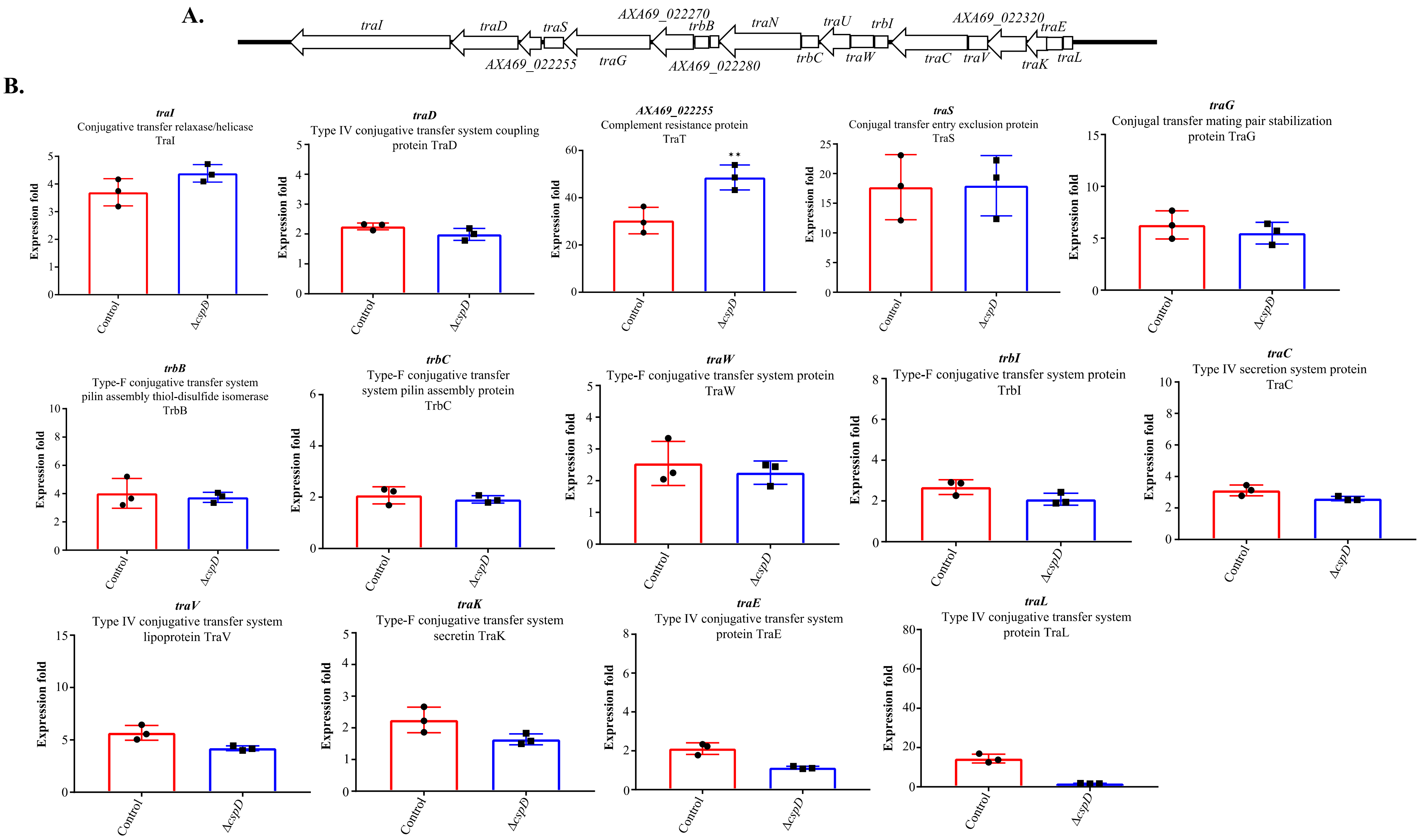

Figure S11. Transcripts per million values (TPMs) plots of transfer genes (*tra* operon) located in pASal5 in *A. salmonicida* Δ*cspD* grown in 15 °C. A. Genetic map of transfer related genes located in pASal5; **B.** TPMs expression folds of significant 14 DEGs in Δ*cspD* mutant vs wild type (*$p \leq 0.05$, **$p \leq 0.01$, ***$p \leq 0.001$, ****$p \leq 0.0001$, The non-parametric Kruskal-Wallis test, followed by Dunn's multiple post-hoc test, was done to determine the significant differences).

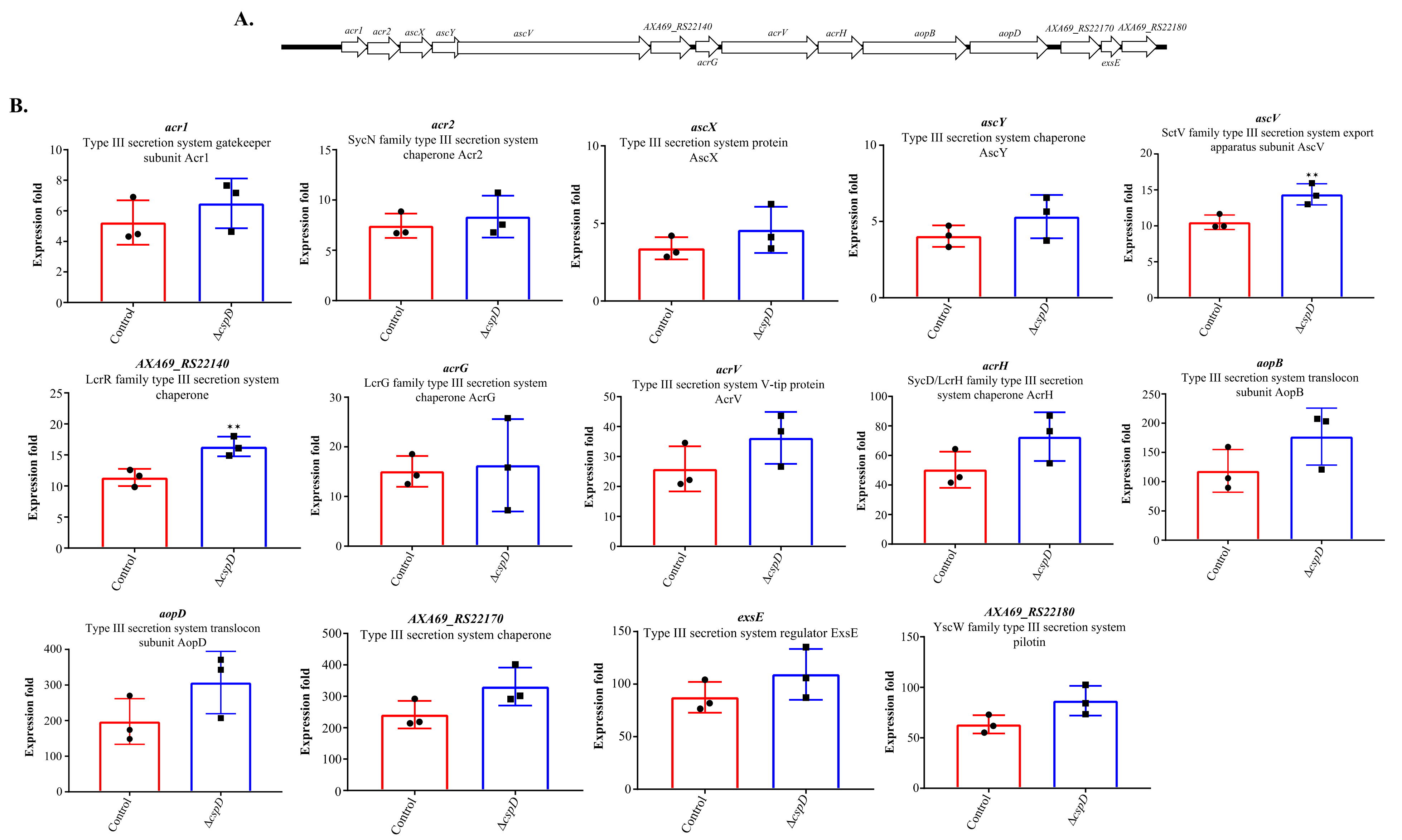

Figure S12. Transcripts per million values (TPMs) plots of T3SS located in pASal5 in *A. salmonicida* Δ*cspD* grown in 15 °C. A. Genetic map of T3SS related genes located in pASal5; **B.** TPMs expression folds of significant 14 DEGs in Δ*cspD* mutant vs wild type (**p < 0.05*, ***p < 0.01*, ****p < 0.001*, *****p < 0.0001*, The non-parametric Kruskal-Wallis test, followed by Dunn's multiple post-hoc test, was done to determine the significant differences).

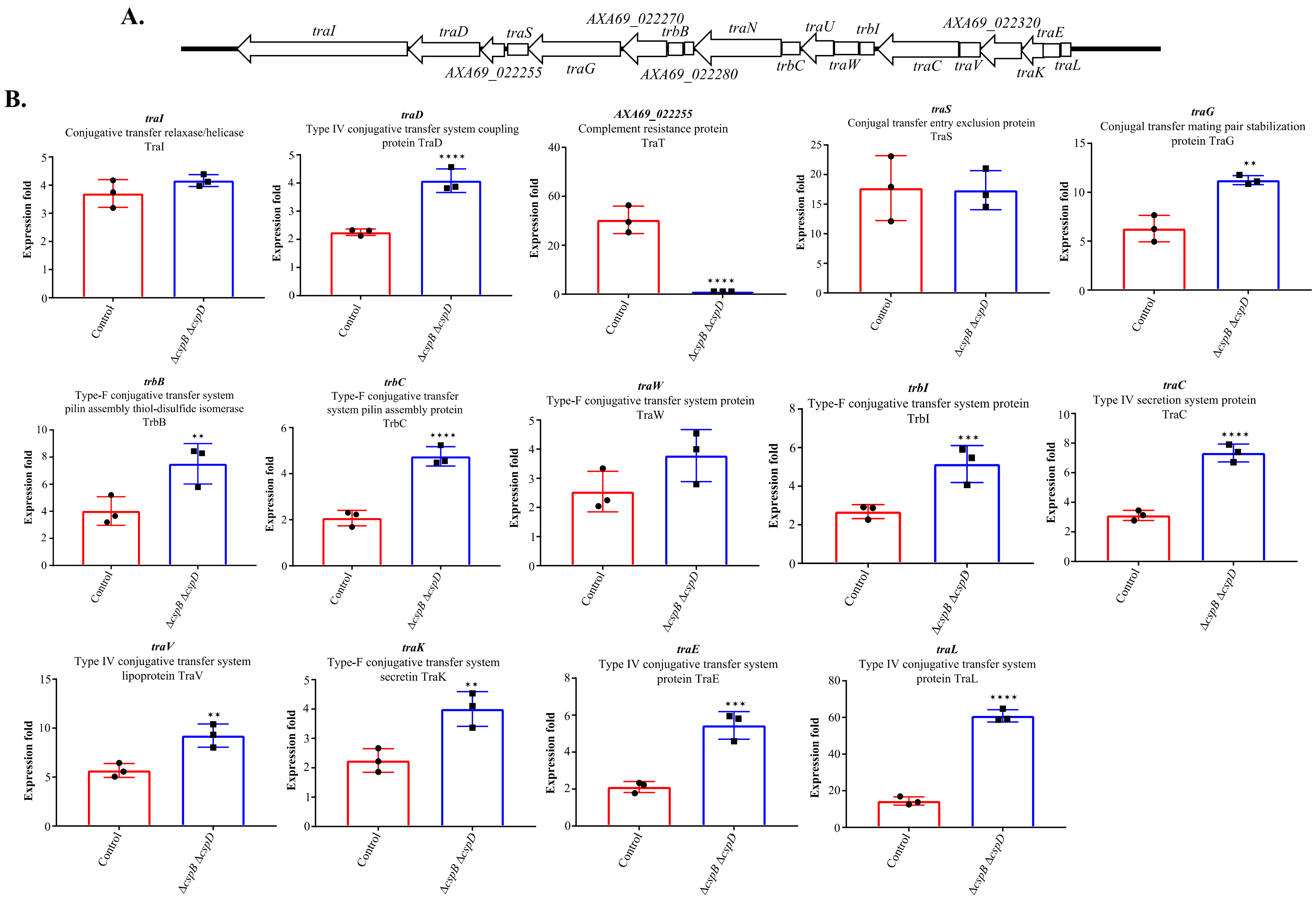

Figure S13. Transcripts per million values (TPMs) plots of transfer genes (*tra* operon) located in pASal5 in *A. salmonicida* Δ*cspB* Δ*cspD* grown in 15 °C. A. Genetic map of transfer related genes located in pASal5; **B.** TPMs expression folds of significant 14 DEGs in Δ*cspB* Δ*cspD* mutant vs wild type (*$p \leq 0.05$, **$p \leq 0.01$, ***$p \leq 0.001$, ****$p \leq 0.0001$, The non-parametric Kruskal-Wallis test, followed by Dunn's multiple post-hoc test, was done to determine the significant differences).

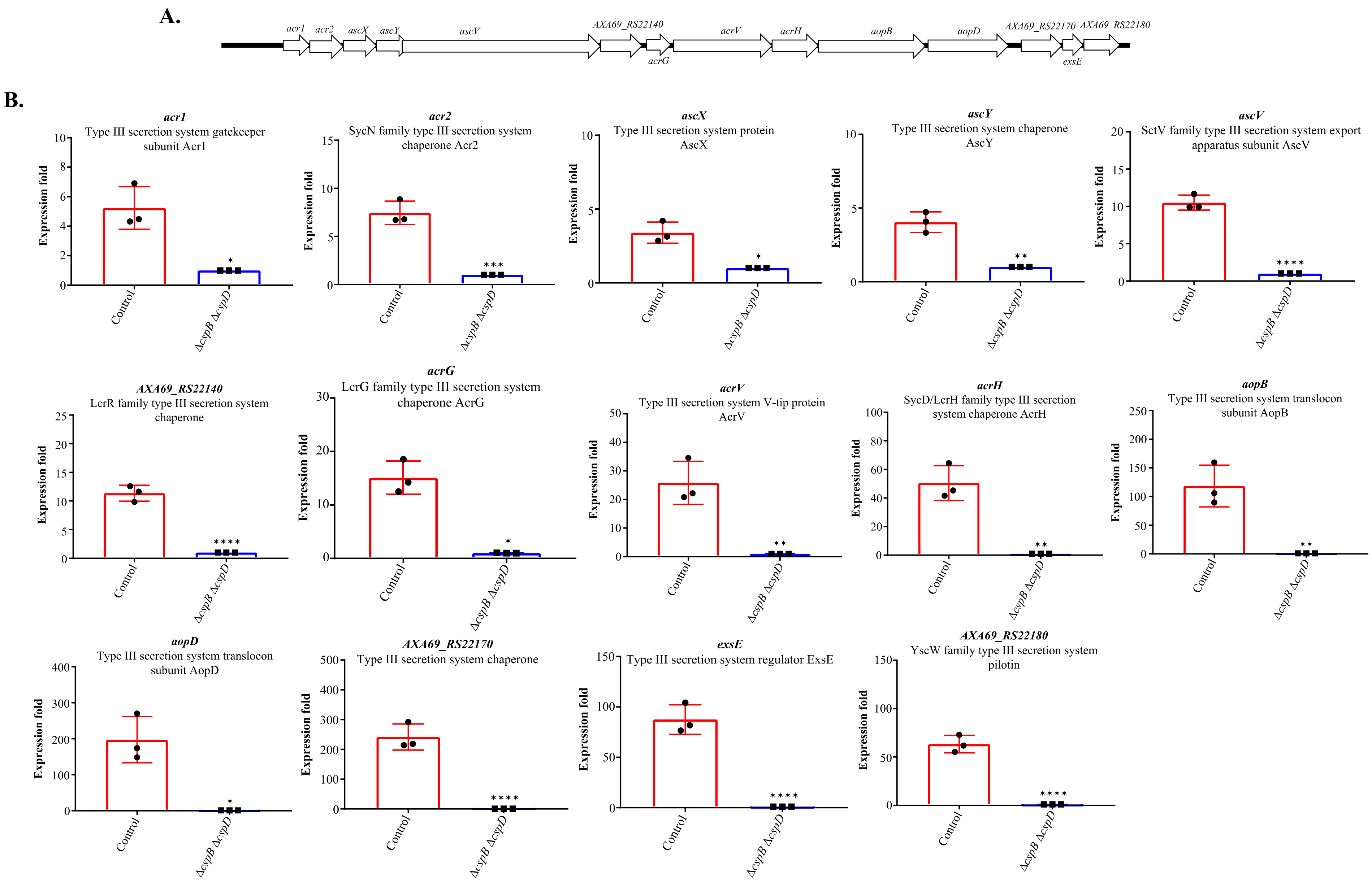

Figure S14. Transcripts per million values (TPMs) plots of T3SS located in pASal5 in *A. salmonicida* Δ*cspB* Δ*cspD* grown in 15°C. A. Genetic map of T3SS related genes located in pASal5; **B.** TPMs expression folds of significant 14 DEGs in Δ*cspB* mutant vs wild type (*$p \leq 0.05$, **$p \leq 0.01$, ***$p \leq 0.001$, ****$p \leq 0.0001$, The non-parametric Kruskal-Wallis test, followed by Dunn's multiple post-hoc test, was done to determine the significant differences).

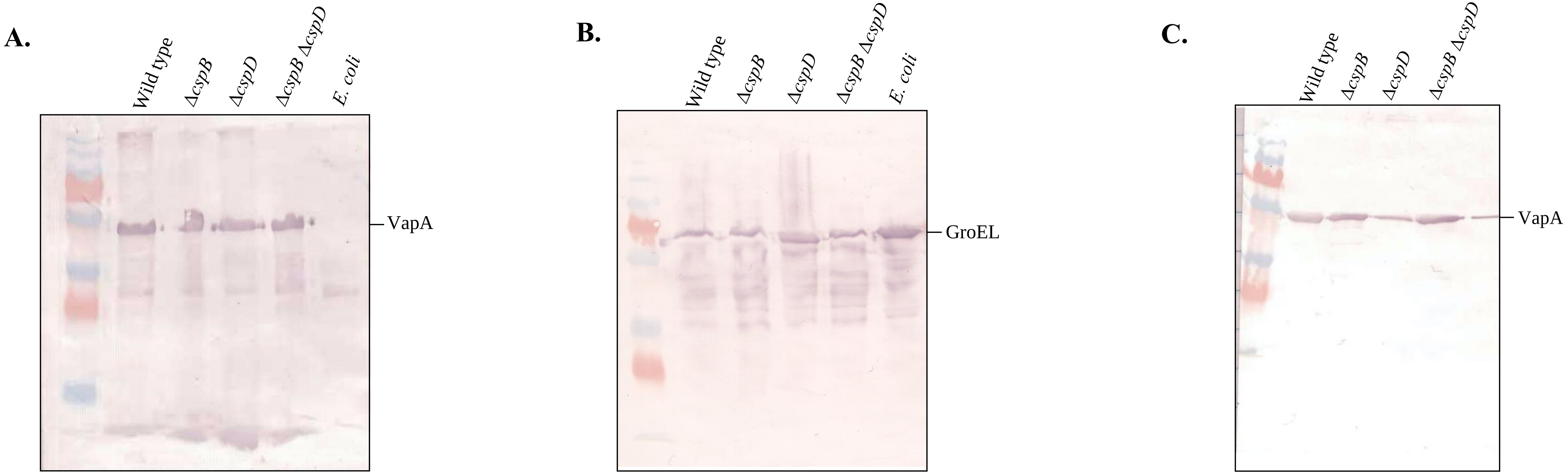

Figure S15. Western blot analysis in *A. salmonicida* strains; **A.** VapA western blot analysis in *A. salmonicida* wild type, Δ*cspB*, Δ*cspD*, and Δ*cspB* Δ*cspD* strains whole cells; **B.** GroEL western blot analysis in *A. salmonicida* wild type, Δ*cspB*, Δ*cspD*, and Δ*cspB* Δ*cspD* strains whole cells; **C.** VapA western blot analysis in *A. salmonicida* wild type, Δ*cspB*, Δ*cspD*, and Δ*cspB* Δ*cspD* OMPs. (e.g., whole blot image of the figure 3B and 3D)
