# Supplementary material for: Role of cold shock proteins B and D in Aeromonas salmonicida subsp. salmonicida physiology and virulence in lumpfish (Cyclopterus lumpus)
Source: Infect Immun. 2024 Jun 26;92(8):e00011-24. doi: 10.1128/iai.00011-24 (PMC11320987; doi:10.1128/iai.00011-24)
Supplement: Supplemental tables — Tables S1 to S4. [file iai.00011-24-s0004.docx]

**Table S1. Primers utilized in this study.**

| **Oligonucleotide** | **Sequence (3´- 5´)** |
| --- | --- |
| Δ*cspB* *A.sal* F1 (*SphI*) | ACATGCATGC CGGTCGGGTAGTGCTGCTCG |
| Δ*cspB* *A.sal* R1 (*XhoI*-*PstI*) | CTCGAGcggAAAACTGCAGTTTT ACATAGGGATCAGGGGGAAA |
| Δ*cspB* *A.sal* F2 (*XhoI*-*PstI*) | AAAACTGCAGTTTTCCGCTCGAGTGAGGCTGGCCTGCCCGATT |
| Δ*cspB* *A.sal* R2 *(XbaI*) | TCGTCTAGA TAGTGGATAACCCCAGCCCG |
| Δ*cspD* *A.sal* F1 (*SphI*) | ACATGCATGCACATAGGCACCTCCCCTAGT |
| Δ*cspD* *A.sal* R1 (*XhoI*-*PstI*) | CTCGAGCGGAAAACTGCAGTTTTACCGAAGCGCGACTATGAGC |
| Δ*cspD* *A.sal* F2 (*XhoI*-*PstI*) | AAAACTGCAGTTTTCCGCTCGAG TAAGAGTCAAATTGGATAAA |
| Δ*cspD* *A.sal* R2 *(XbaI*) | TCGTCTAGA ACGCTGGAGCTGCTGCTGGC |
| *vapA*-2042F | CCATCTTCGGTCATCAGTGCTGT |
| *vapA*-2042R | AGATAAATCGCAAGGTAGCTAACG |
| *vapA*-Center F | ACTAACCAGACCATCCAACGTGACAAGGTT |
| *vapA*-Center R | AACCTTGTCACGTTGGATGGTCTGGTTAGT |

**Table S2. Concentration of RNA for transcriptomic analysis at 15°C.**

| Strains (OD600nm) | Concentration (µg/mL) | Ratio  (260/280) | Ratio  (260/230) | CFU/mL |
| --- | --- | --- | --- | --- |
| Wild Type _(.720)_ | 729.79 | 2.06 | 1.8 | 9.8 x10^8^ |
| Wild Type _(.760)_ | 720.74 | 2.09 | 1.7 | 9.2 x10^8^ |
| Wild Type _(.825)_ | 376.24 | 2.04 | 1.5 | 7.7 x10^8^ |
| Δ*cspB* _(.810)_ | 3370.2 | 2.07 | 2.1 | 6.2 x10^8^ |
| Δ*cspB* _(.776)_ | 1421.9 | 2.06 | 1.6 | 8.1 x10^8^ |
| Δ*cspB* _(.736)_ | 1050.1 | 2.07 | 1.7 | 8.8 x10^8^ |
| Δ*cspD* _(.709)_ | 452.15 | 2.06 | 1.6 | 8.1 x10^8^ |
| Δ*cspD* _(.789)_ | 590.93 | 2.04 | 1.8 | 9.8 x10^8^ |
| Δ*cspD* _(.781)_  Δ*cspB* Δ*cspD* _(.764)_  Δ*cspB* Δ*cspD* _(.751)_  Δ*cspB* Δ*cspD* _(.725)_ | 2191.4  3604.1  2084.3  1569.4 | 2.09  2.00  2.00  2.00 | 2.0  2.1  2.2  2.1 | 7.0 x10^8^  5.7 x10^8^  6.3 x10^8^  6.2 x10^8^ |

**Table S3. Biochemical profiling of *A. salmonicida* wild type and mutants using API 20E.**

| **Biochemicals/Enzymes** | **Wild type** | **∆*cspB*** | **∆*cspD*** | **∆*cspB* ∆*cspD*** |
| --- | --- | --- | --- | --- |
| Nitrates to nitrogen | + | + | + | + |
| Indole production | - | - | - | - |
| Acidification | + | + | - | + |
| Arginine dihydrolase | + | - | + | - |
| Urease | - | - | - | - |
| Hydrolysis (β-glucosidase) | + | + | + | + |
| Hydrolysis (protease) | + | + | + | + |
| β-galactosidase | - | - | - | - |
| Glucose assimilation | - | + | - | + |
| Arabinose assimilation | - | - | - | - |
| Mannose assimilation | - | - | - | - |
| Mannitol assimilation | - | + | - | + |
| N-acetyl-glucosamine assimilation | - | + | - | + |
| Maltose assimilation | + | + | - | + |
| Gluconate assimilation | - | - | - | - |
| Caprate assimilation | - | - | - | - |
| Adipate assimilation | - | - | - | - |
| Malate assimilation | + | + | - | + |
| Citrate assimilation | - | - | - | - |
| Phenyl-acetate assimilation | - | - | - | - |

**Table S4. Transcriptomics mapping statistics.**

| **Samples** | **Reads** | **Reads after trimming** | **% Trimmed**  **reads** | **Mapped reads** | **Mapped genes** | **% Mapped reads** |
| --- | --- | --- | --- | --- | --- | --- |
| Control _1_ | 146,551,000 | 146,533,334 | 99.99 | 142,646,315 | 37,522,140 | 96.26 |
| Control _2_ | 176,735,710 | 176,705,804 | 99.98 | 172,744,942 | 46,567,872 | 96.91 |
| Control _3_ | 98,472,366 | 98,443,329 | 99.97 | 96,929,199 | 27,729,305 | 97.75 |
| Δ*cspB*_1_ | 143,318,698 | 142,039,090 | 99.11 | 138,007,750 | 58,927,371 | 96.60 |
| Δ*cspB*_2_ | 101,651,214 | 100,554,365 | 98.92 | 98,188,174 | 41,793,186 | 97.35 |
| Δ*cspB*_3_ | 73,560,508 | 73,010,131 | 99.25 | 71,295,459 | 30,786,343 | 97.24 |
| Δ*cspD*_1_ | 66,726,830 | 66,134,251 | 99.11 | 64,987,322 | 27,518,119 | 97.98 |
| Δ*cspD*_2_ | 83,508,032 | 82,691,849 | 99.02 | 80,993,660 | 34,386,569 | 97.45 |
| Δ*cspD*_3_ | 153,313,584 | 152,076,426 | 99.19 | 149,197,793 | 64,969,822 | 97.72 |
| Δ*cspB* Δ*cspD*_1_ | 149,800,466 | 148,657,157 | 99.24 | 145,463,793 | 57,951,169 | 97.35 |
| Δ*cspB* Δ*cspD*_2_ | 134,318,572 | 133,355,405 | 99.28 | 130,548,905 | 52,424,044 | 97.38 |
| Δ*cspB* Δ*cspD*_3_ | 139,533,914 | 138,195,834 | 99.04 | 135,237,831 | 54,825,489 | 97.43 |
